# Supplementary material for: Designing tailored combinations of structural units in polymer dielectrics for high-temperature capacitive energy storage
Source: Nat Commun. 2023 Apr 26;14:2406. doi: 10.1038/s41467-023-38145-w (PMC10133333; doi:10.1038/s41467-023-38145-w)
Supplement: Supplementary file 1 — Supplementary Information [file 41467_2023_38145_MOESM1_ESM.pdf]

Supplementary Information

for

Designing tailored combinations of structural units in polymer dielectrics for high-temperature capacitive energy storage

Wang et al.

Section 1. Structure characterization

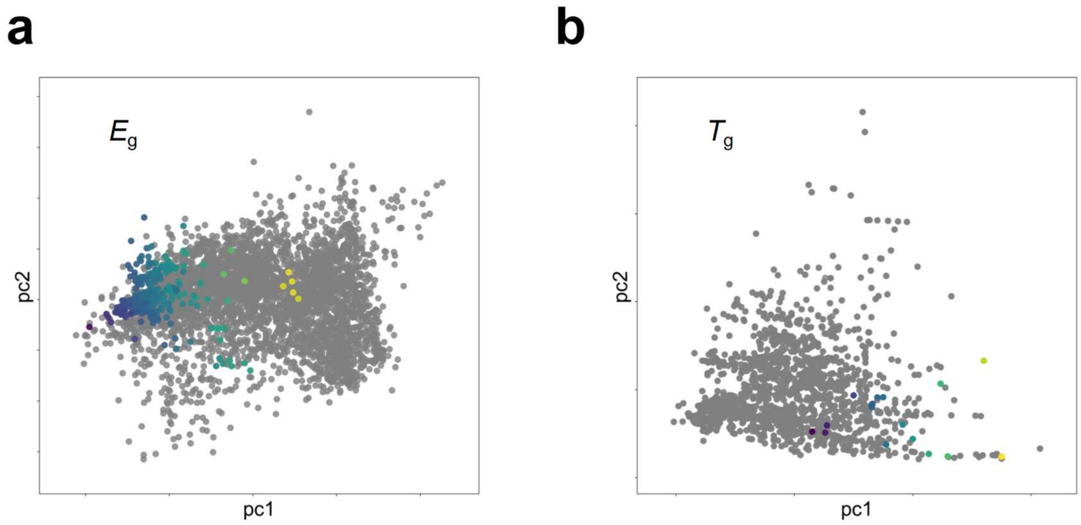

Supplementary Figure S1 Chemical space of (a) E_g training set (4208 polymers) and (b) T_g training set (2988 polymers) (gray circles) considered the PI system polymer (highlighted), illustrated using the first two principal components (pc1 and pc2).

Supplementary Table S1. Coordinates for combinations of polymers shown in Figure 2e.

Structure	E_g (eV)	T_g (°C)
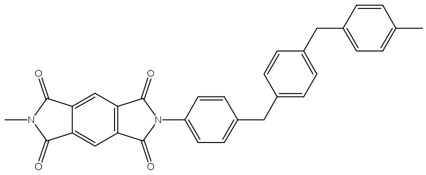	2.7	324
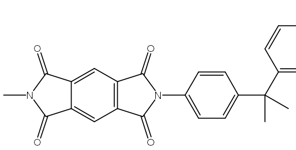	2.8	340
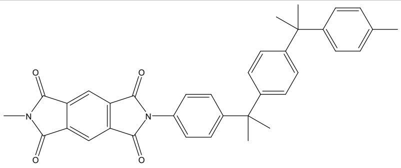	3.3	315
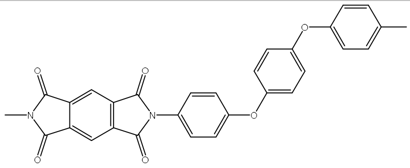	2.5	300
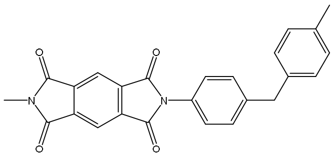	2.3	337
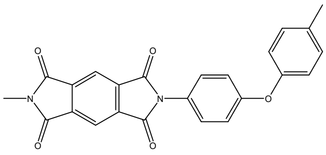	2.2	362
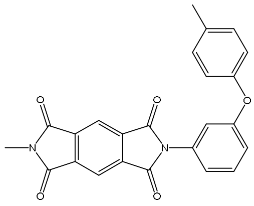	2.2	348

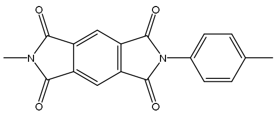	1.6	322
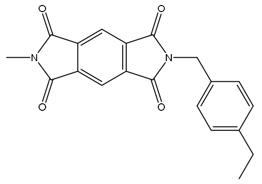	2.6	309
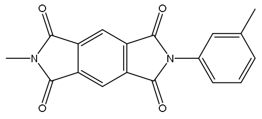	1.6	353
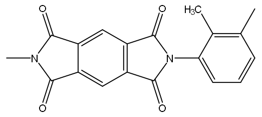	1.9	389
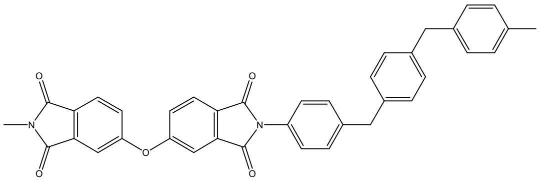	3.0	279
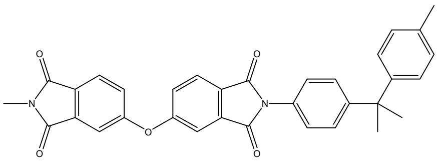	3.0	280
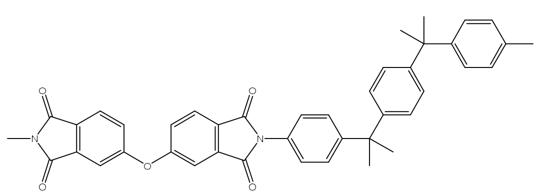	3.5	262
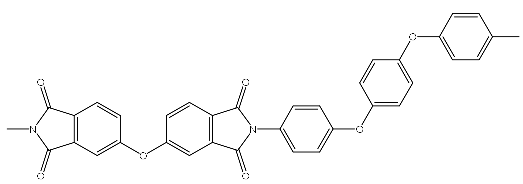	2.8	242
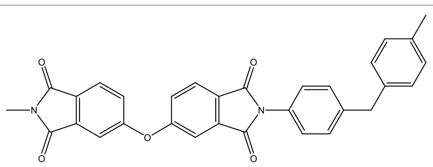	2.7	286

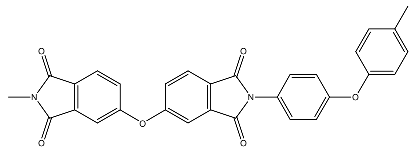	2.6	263
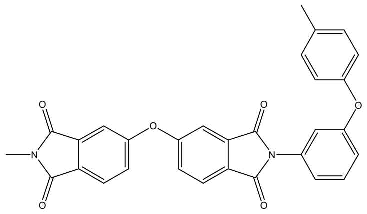	2.6	263
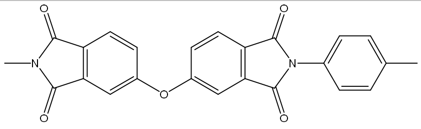	2.2	295
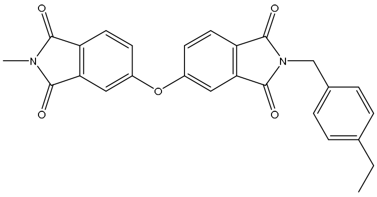	3.1	253
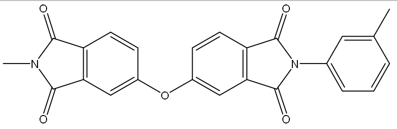	2.2	295
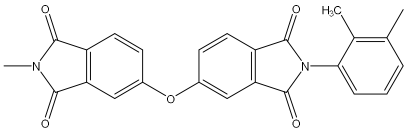	2.4	277
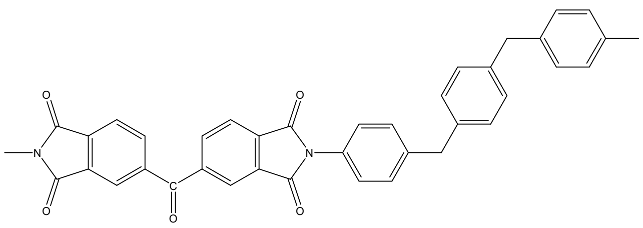	2.3	298
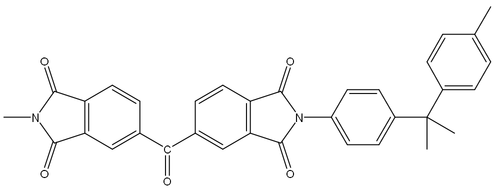	2.3	306

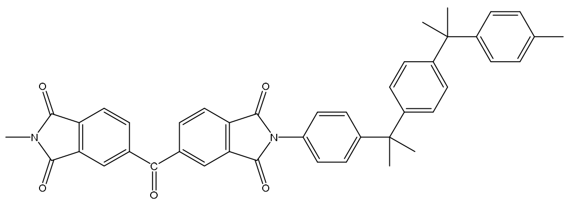	2.8	280
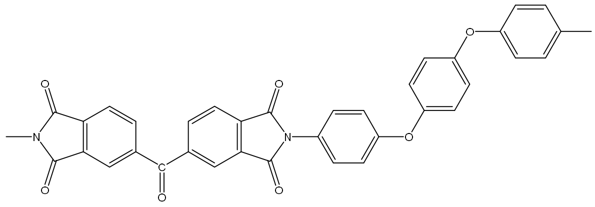	2.1	270
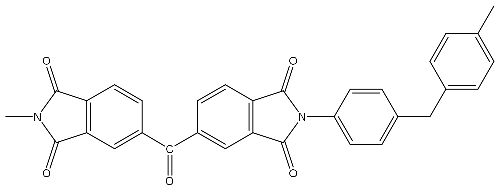	1.9	308
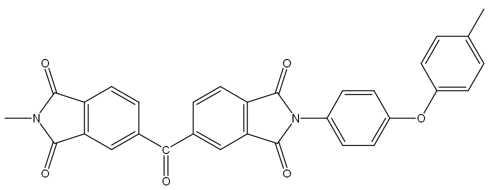	1.8	290
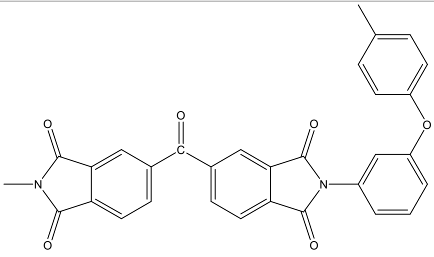	1.8	292
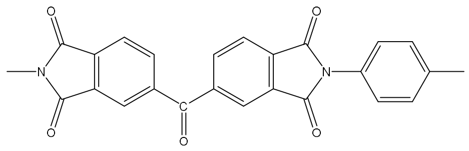	1.4	309
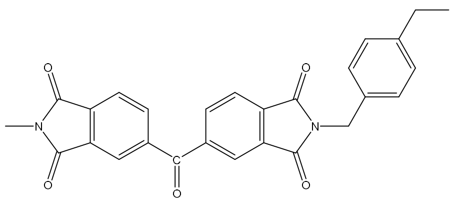	2.3	290
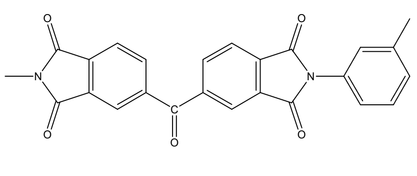	1.4	320

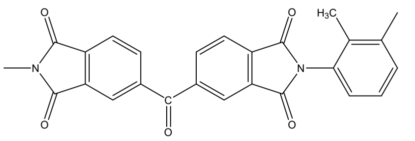	1.6	290
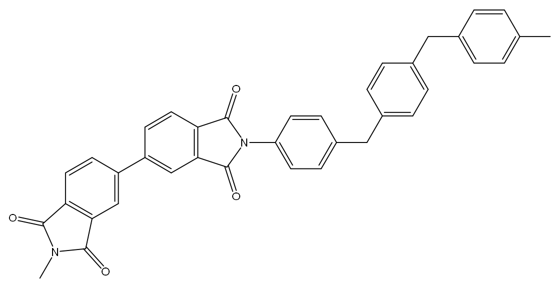	2.4	307
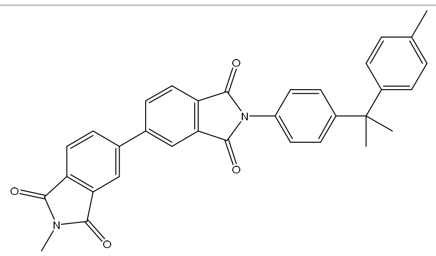	2.5	312
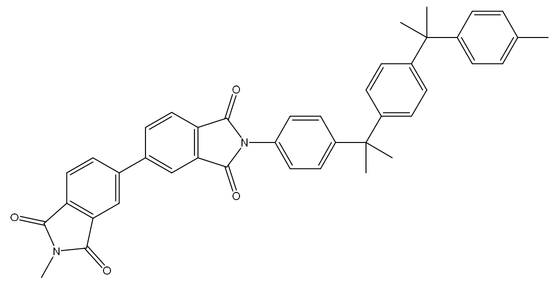	3.0	282
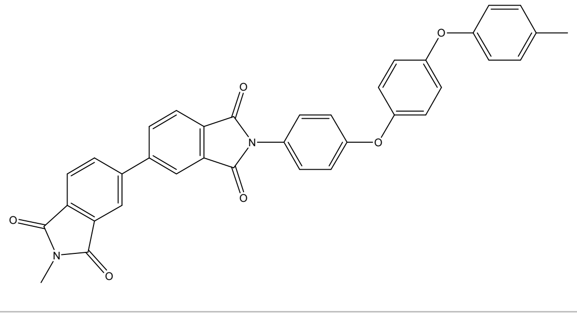	2.2	270
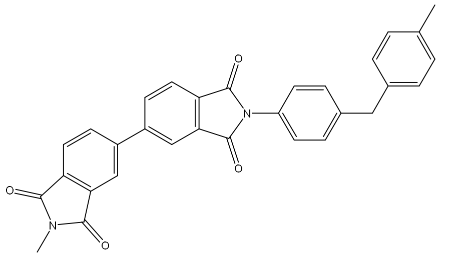	2.1	324

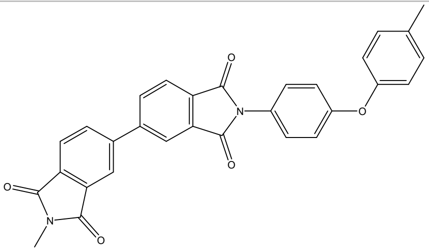	1.9	300
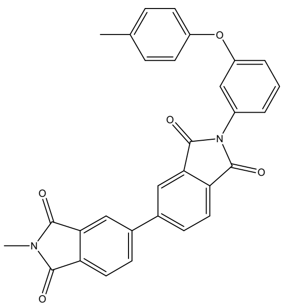	1.9	295
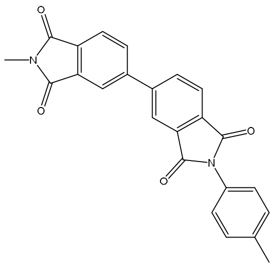	1.5	343
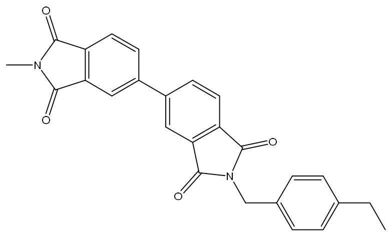	2.4	297
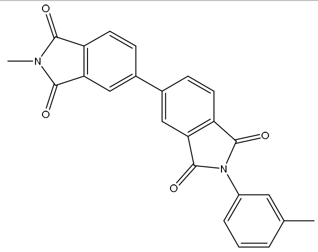	1.5	337
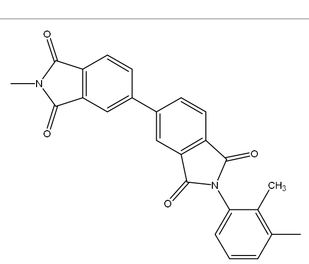	1.7	337

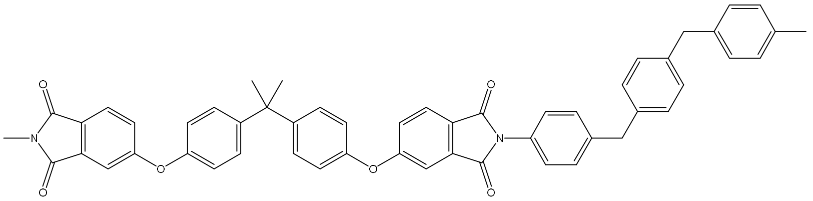	3.1	238
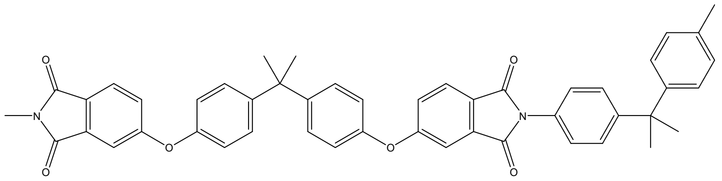	3.2	234
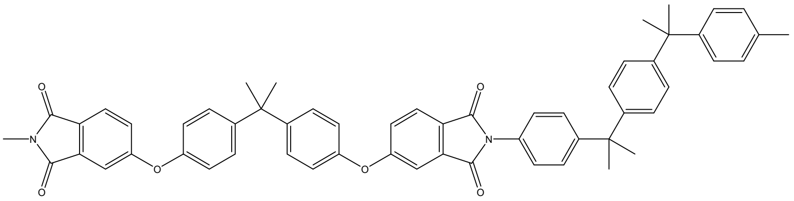	3.4	221
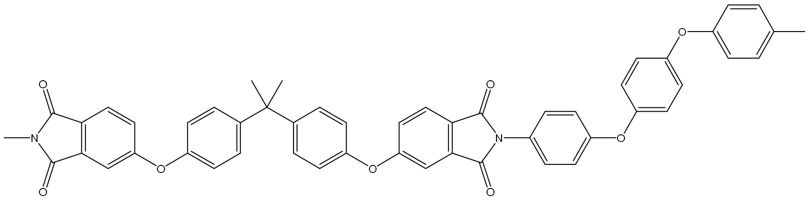	3.0	222
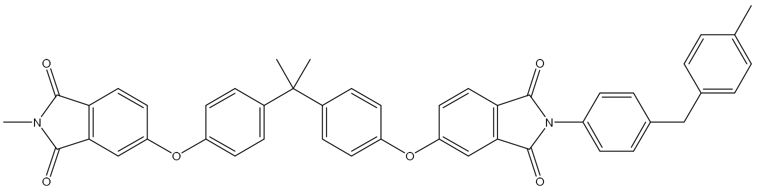	3.0	241
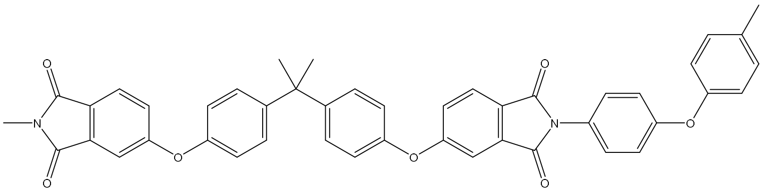	3.0	231
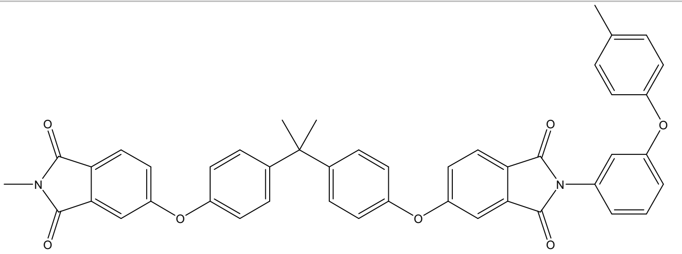	3.0	221
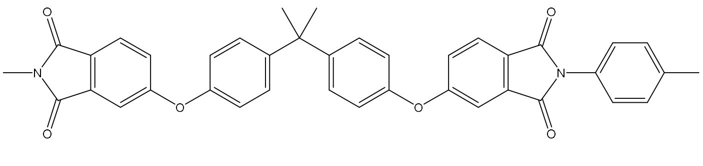	2.9	243

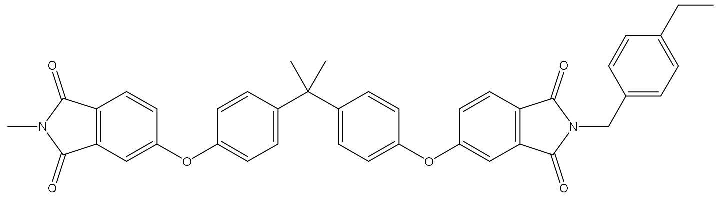	3.3	220
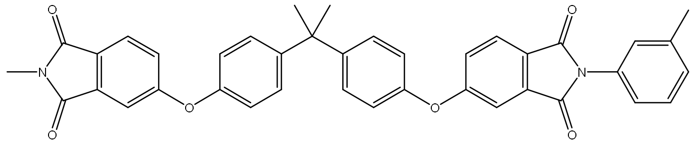	2.9	235
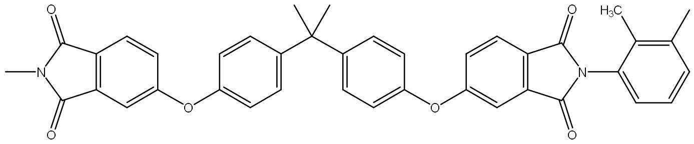	2.9	229
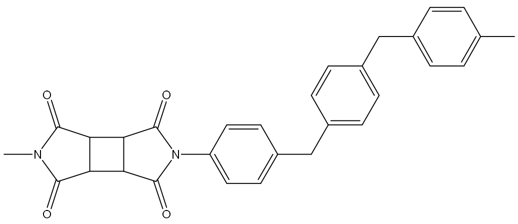	3.5	280
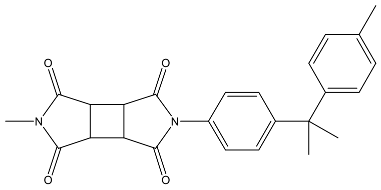	3.6	266
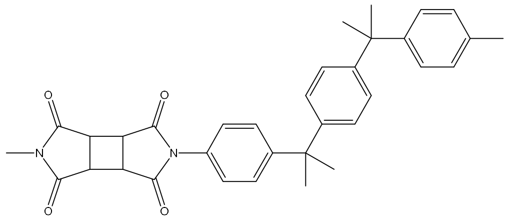	3.9	260
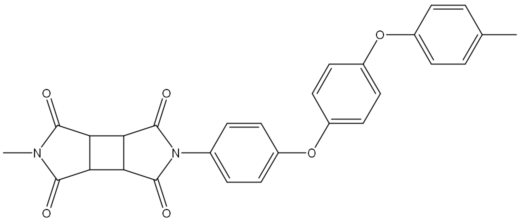	3.4	243

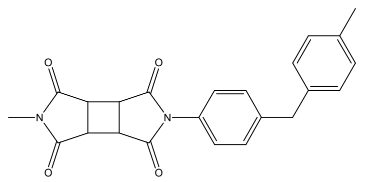	3.4	279
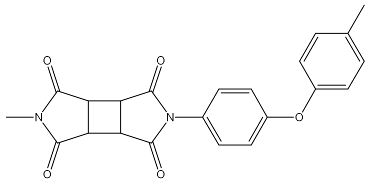	3.3	256
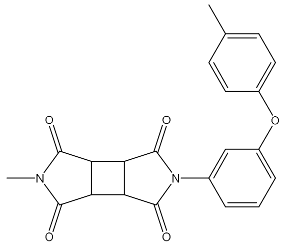	3.2	260
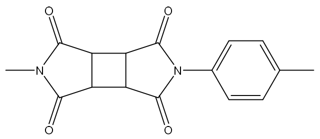	3.0	251
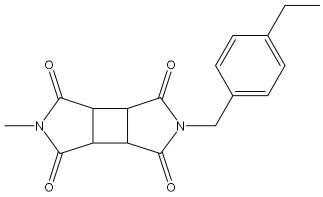	4.0	208
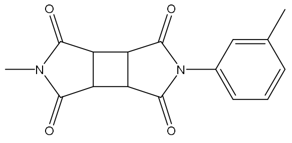	3.0	255
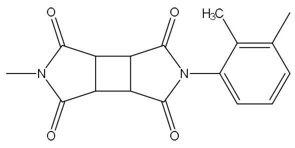	3.2	248
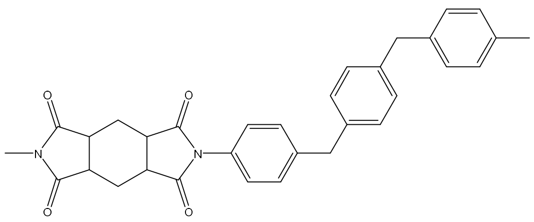	3.7	290

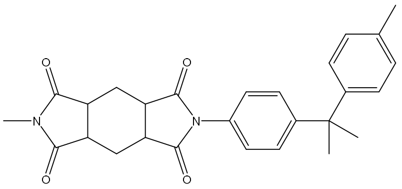	3.8	284
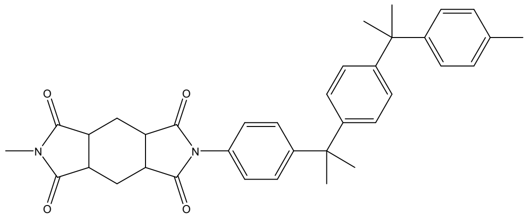	4.0	274
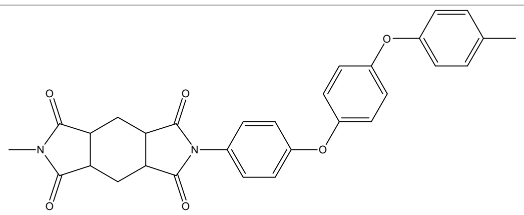	3.6	258
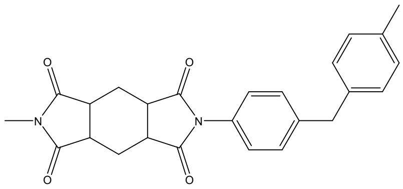	3.6	294
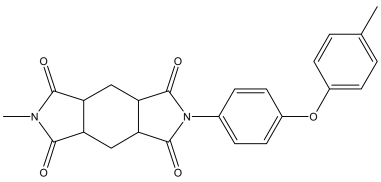	3.5	279
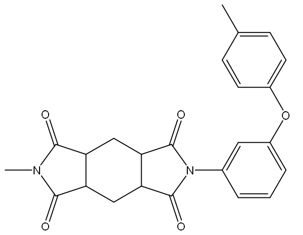	3.5	271
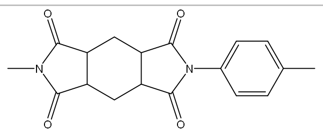	3.4	278
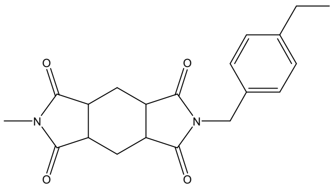	4.2	240

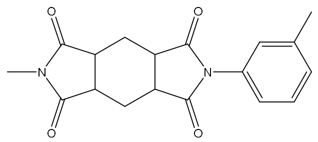	3.4	273
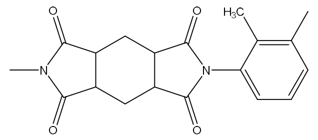	3.5	292
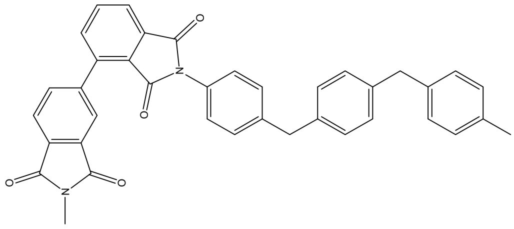	2.7	279
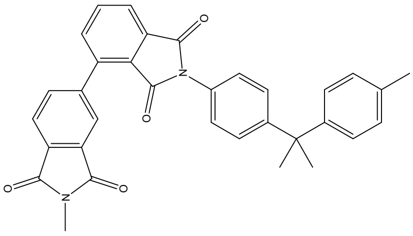	2.7	280
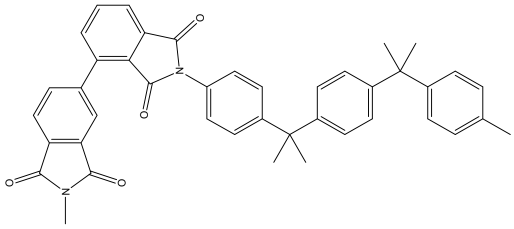	3.2	262
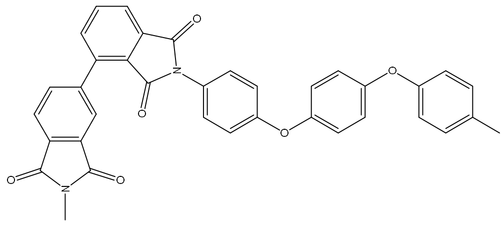	2.5	242
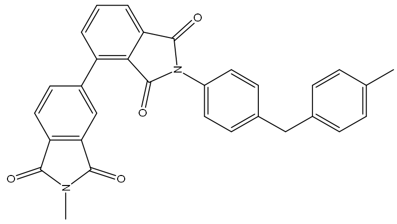	2.4	286

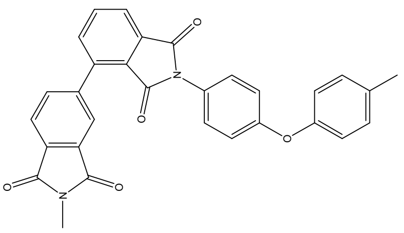	2.3	263
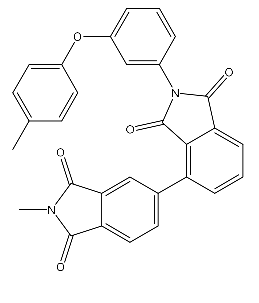	2.3	263
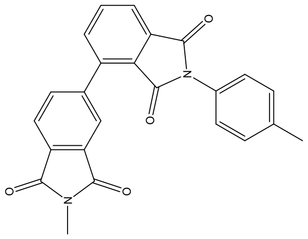	1.9	295
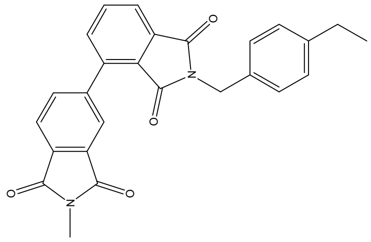	2.8	253
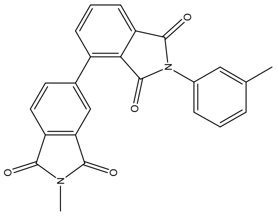	1.9	295
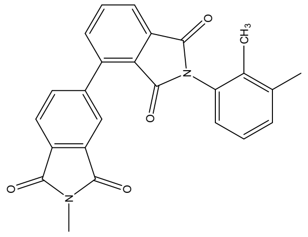	2.1	277

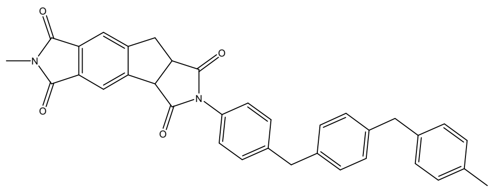	3.5	272
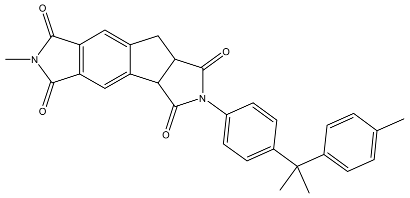	3.6	253
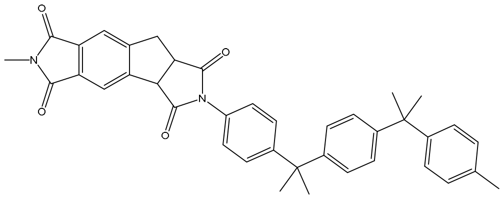	3.8	255
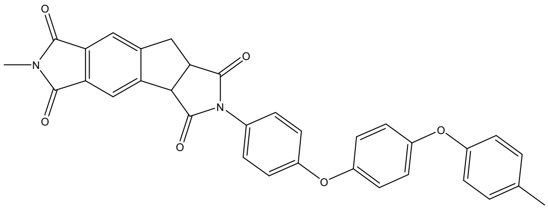	3.4	239
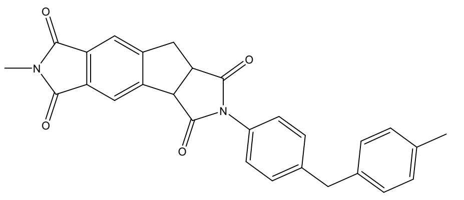	3.4	263
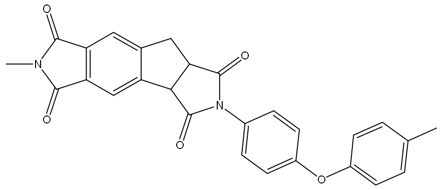	3.3	244
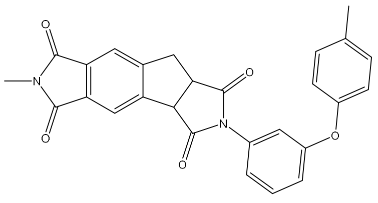	3.3	249

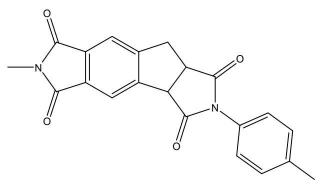	3.1	234
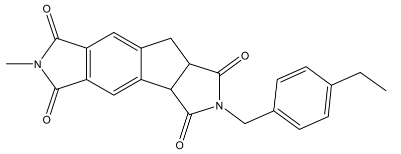	4.0	194
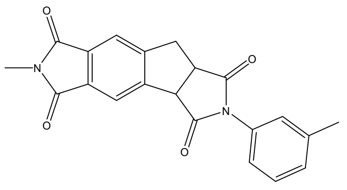	3.1	240
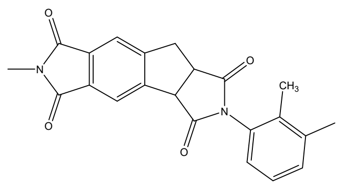	3.3	288
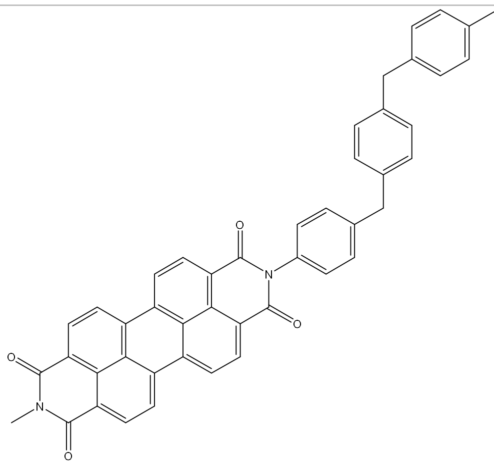	1.9	324
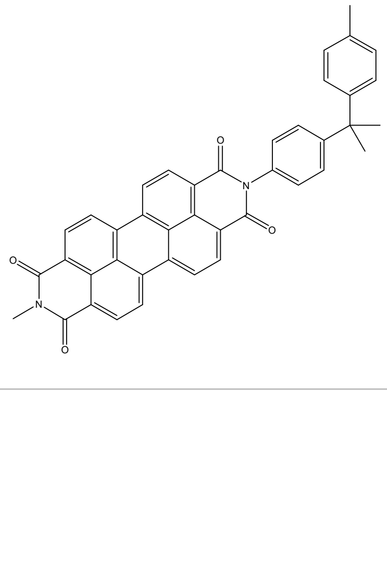	1.9	337

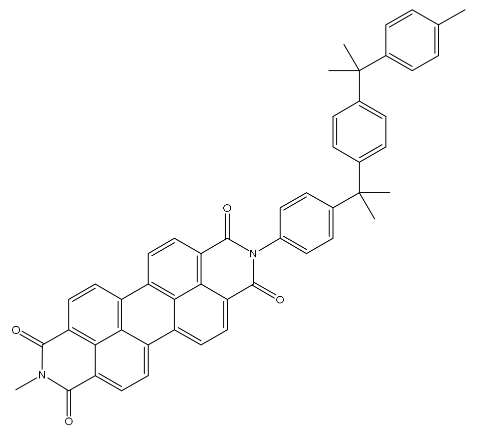	2.5	320
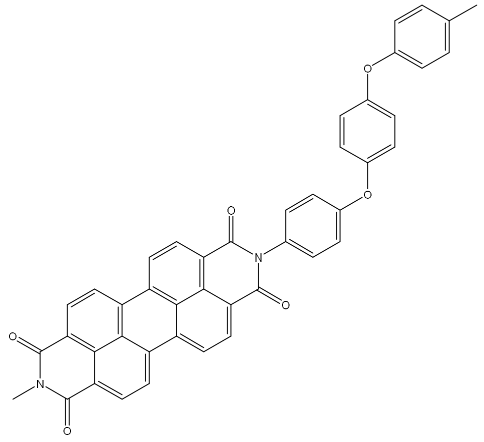	1.7	303
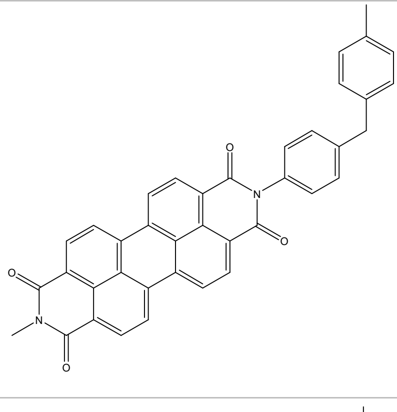	1.4	339
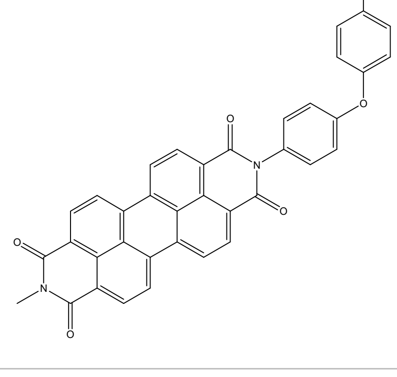	1.3	328

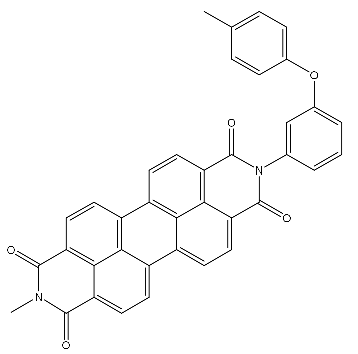	1.3	323
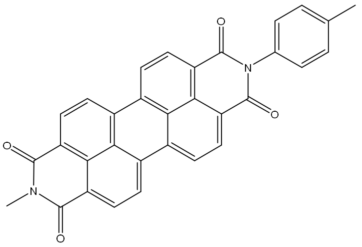	0.8	352
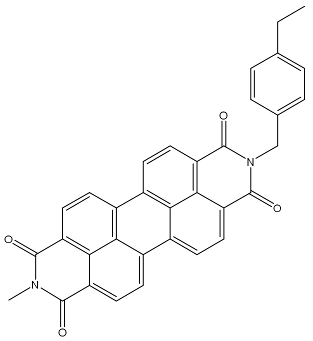	1.6	333
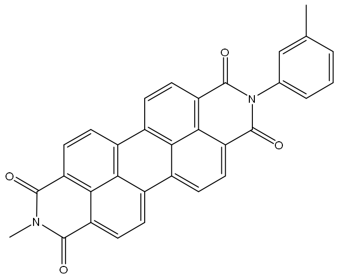	0.8	347
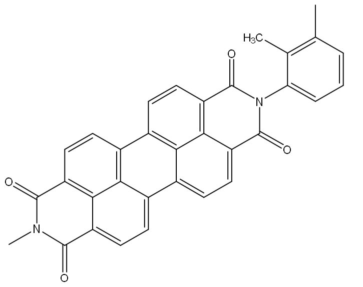	1.1	393

1

PI (A6-B1)

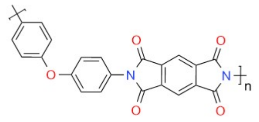

2

PI-*m* (A7-B1)

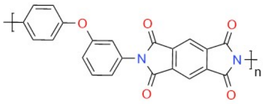

3

PI-methyl (A5-B1)

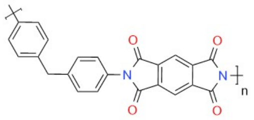

4

PI-ether (A4-B1)

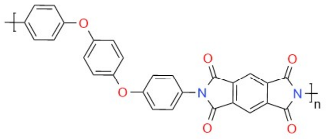

5

PI-iso (A3-B1)

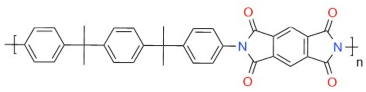

6

PI-oxo (A6-B2)

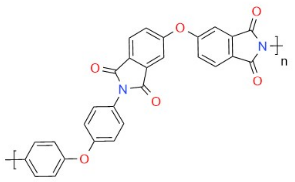

7

PI-oxo-M (A5-B2)

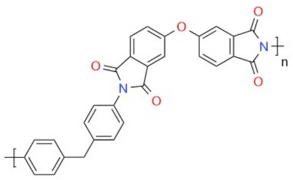

8

PI-oxo-iso (A3-B2)

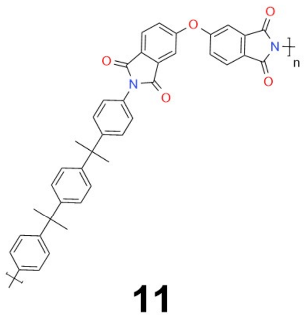

9

PEI (A10-B5)

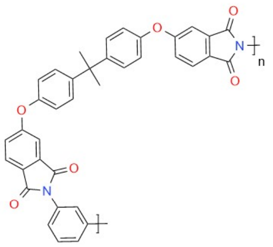

10

PEI-*p* (A8-B5)

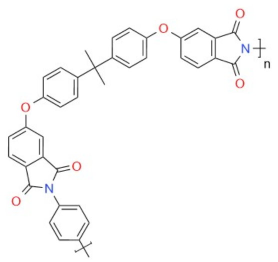

11

PEI-*o*-M (A11-B5)

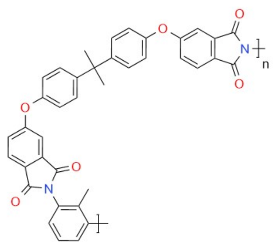

12

PI-cyc-iso (A3-B7)

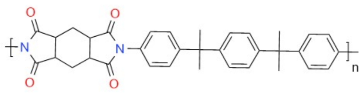

Supplementary Figure S2 Chemical structures of the experimental dielectric polymers in this work.

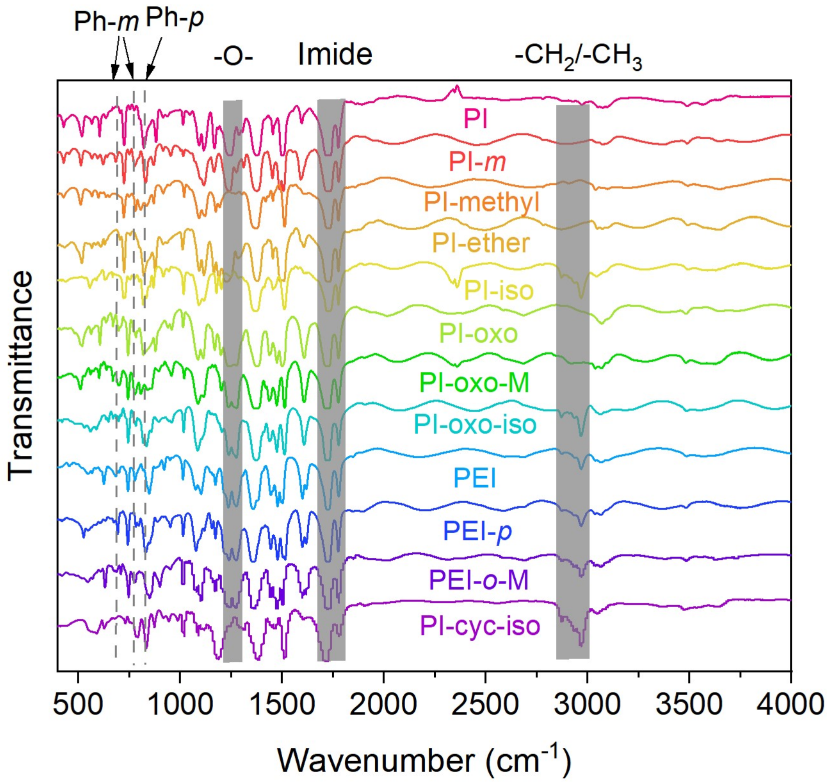

Supplementary Figure S3 ATR-FTIR spectra of the experimental dielectric polymers in this work. Obviously, except for PI-methyl and PI-iso, the characteristic peaks of ether bond (1240 and 1277cm^{-1}) appeared in all the polymers. In addition, compared with PI, PI-*m* has additional characteristic peaks at 680 and 774 cm^{-1}, which can be attributed to *meta*-benzene. On the other hand, all polymers containing isopropyl or methylene structural blocks have characteristic peaks in the range of 2840-2950 cm^{-1}. The above results confirmed the successful synthesis of different polymer molecular structures.

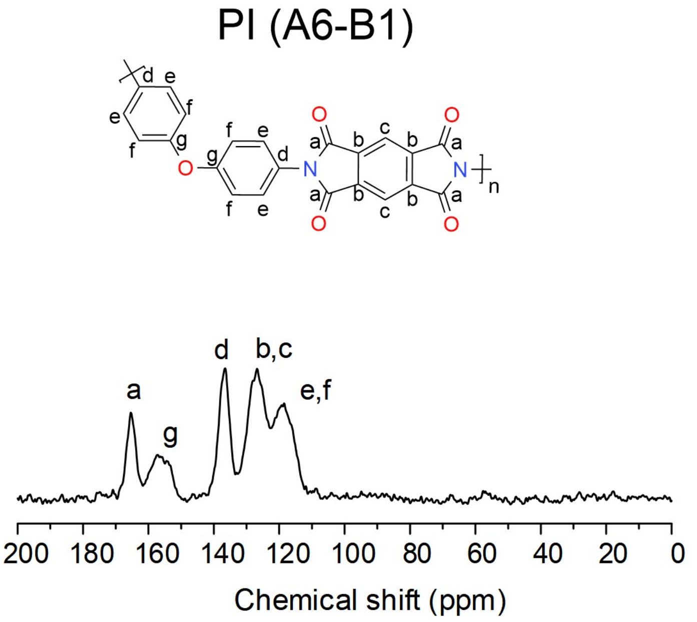

Supplementary Figure S4 ^{13}C NMR spectra of PI.

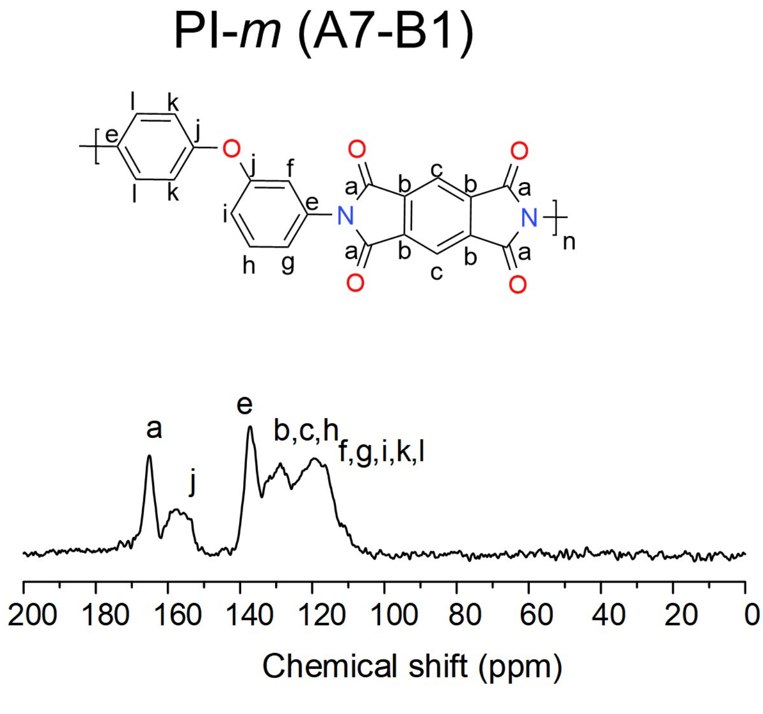

Supplementary Figure S5 ^{13}C NMR spectra of PI-*m*.

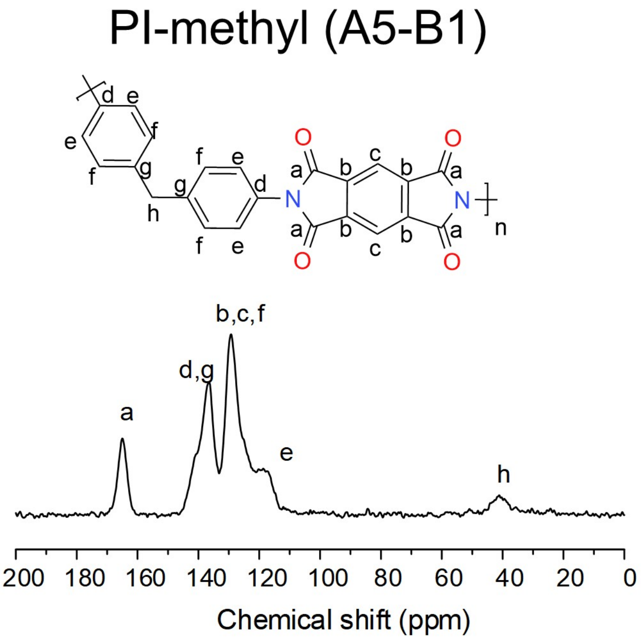

Supplementary Figure S6 ^{13}C NMR spectra of PI-methyl.

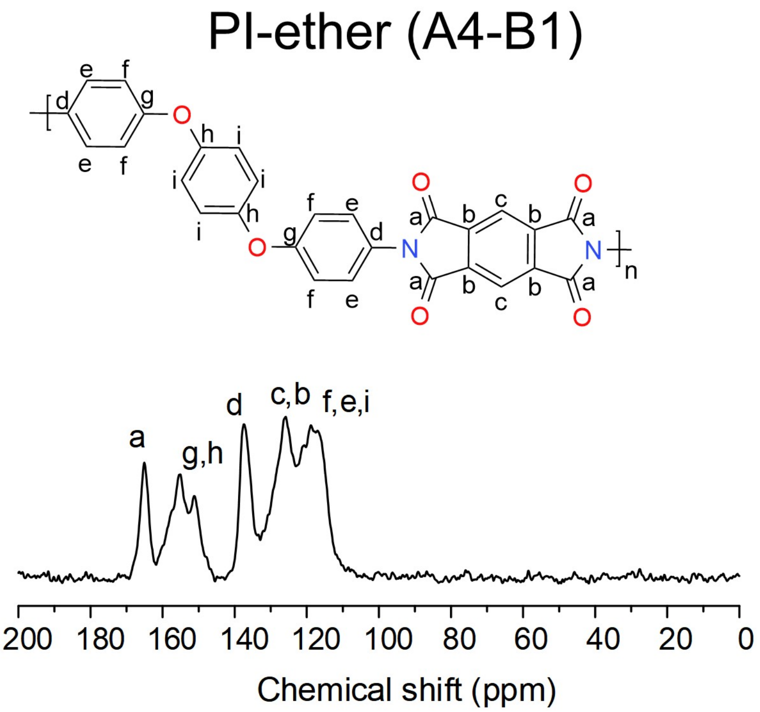

Supplementary Figure S7 ^{13}C NMR spectra of PI-ether.

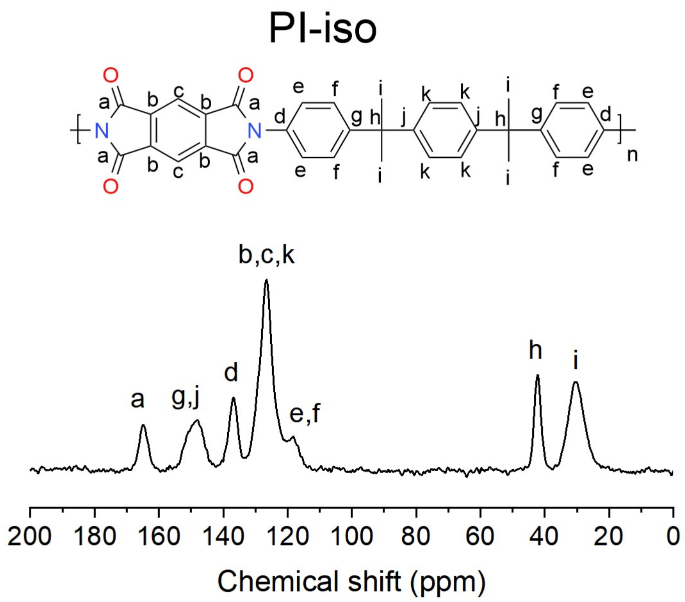

Supplementary Figure S8 ^{13}C NMR spectra of PI-iso.

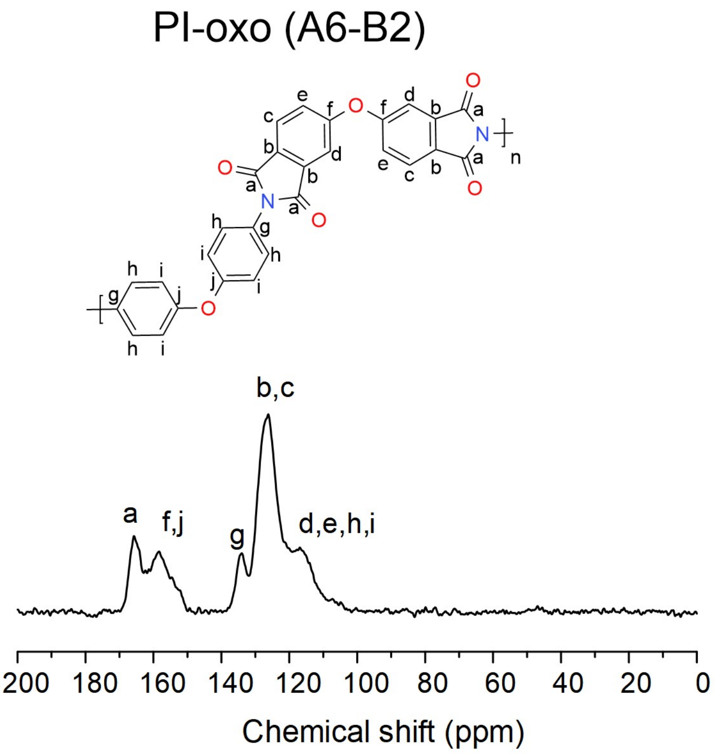

Supplementary Figure S9 ^{13}C NMR spectra of PI-oxo.

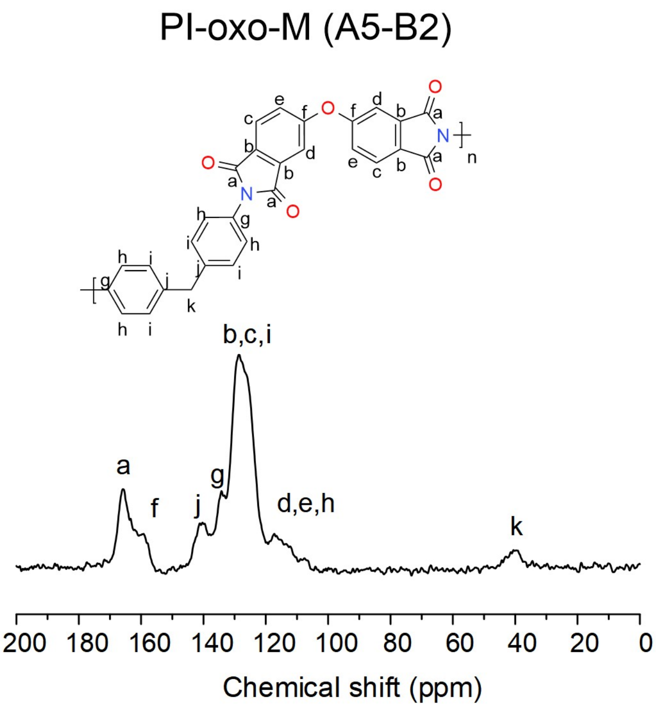

Supplementary Figure S10 ^{13}C NMR spectra of PI-oxo-M.

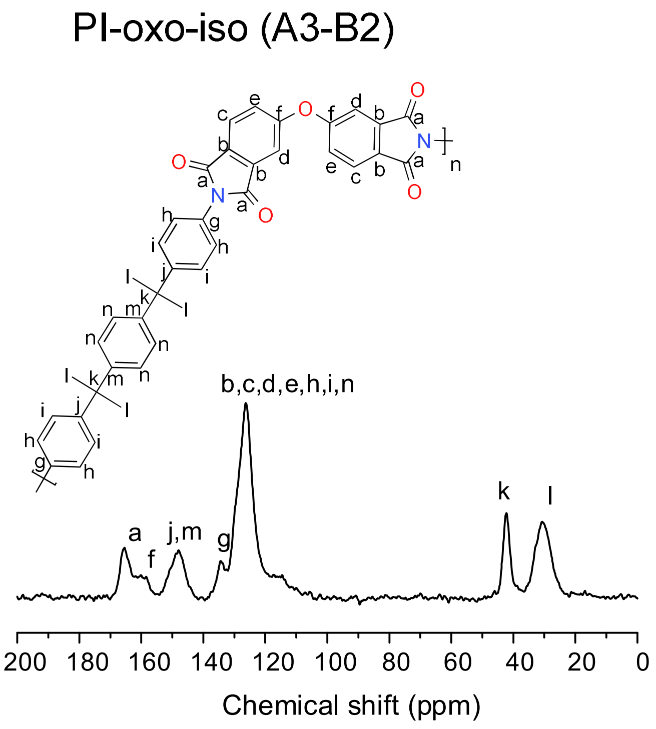

Supplementary Figure S11 ^{13}C NMR spectra of PI-oxo-iso.

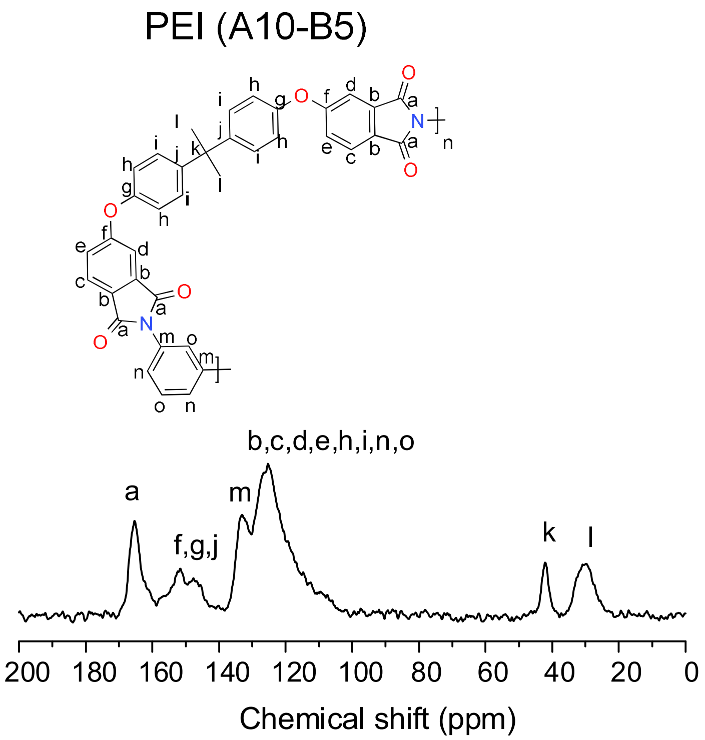

Supplementary Figure S12 ^{13}C NMR spectra of PEI.

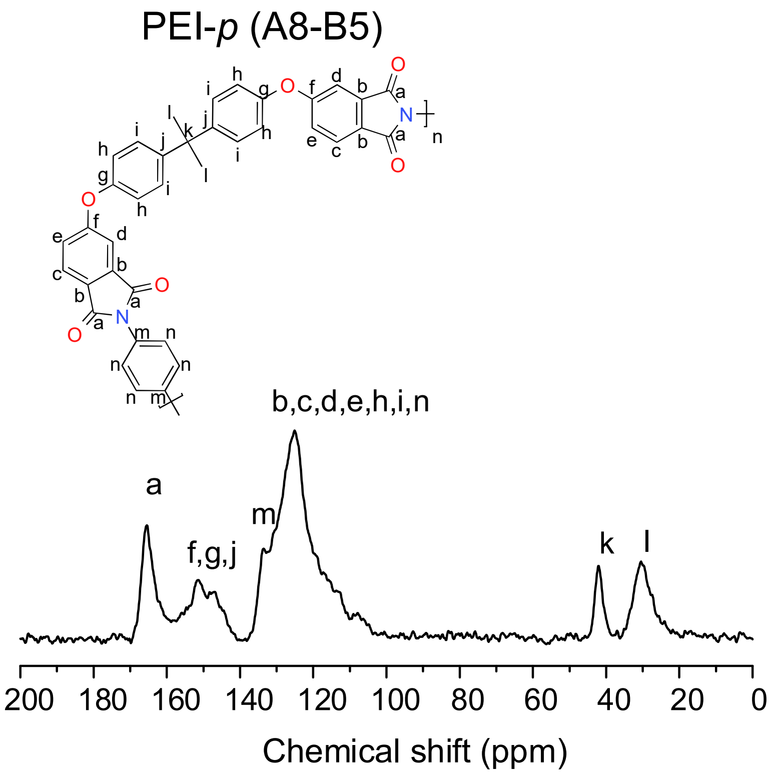

Supplementary Figure S13 ^{13}C NMR spectra of PEI-*p*.

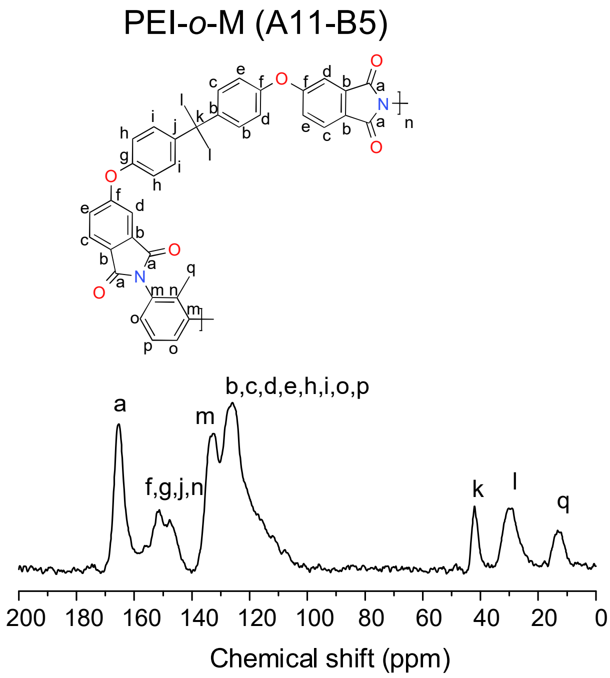

Supplementary Figure S14 ^{13}C NMR spectra of PEI-*o*-M.

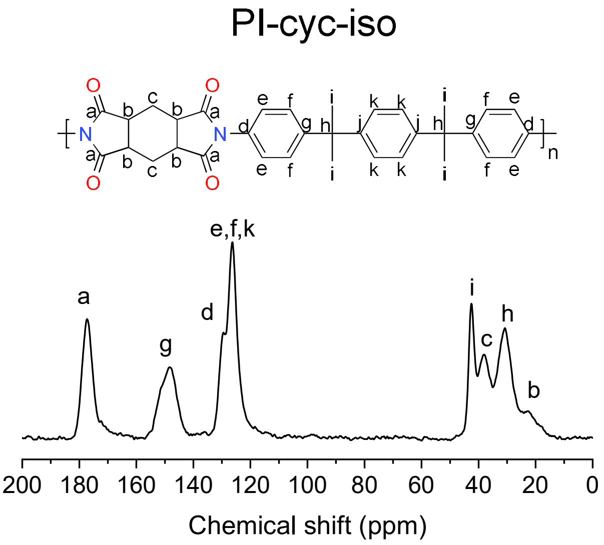

Supplementary Figure S15 ^{13}C NMR spectra of PI-cyc-iso.

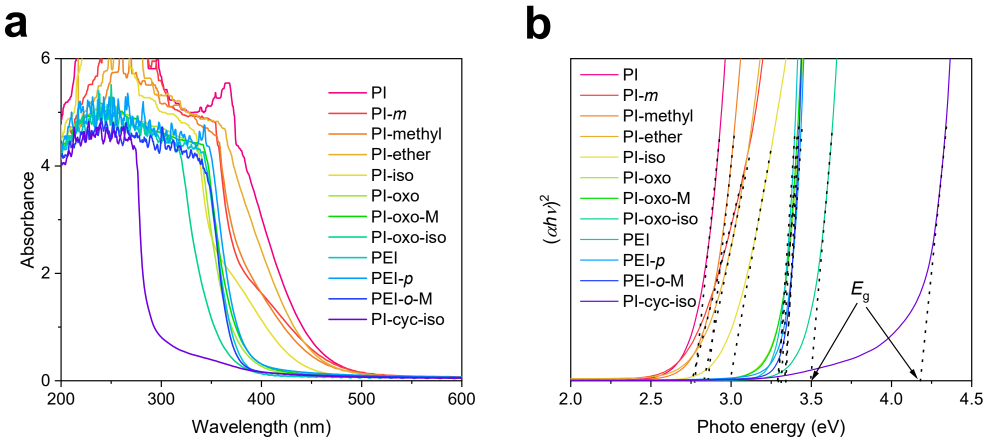

Supplementary Figure S16 (a) UV-vis absorption spectra and (b) $(\alpha hv)^2$-hv plots by Tauc plot of the experimental dielectric polymers in this work, in which α, h, and v are the absorption coefficient, Planck constant, and light frequency, respectively.

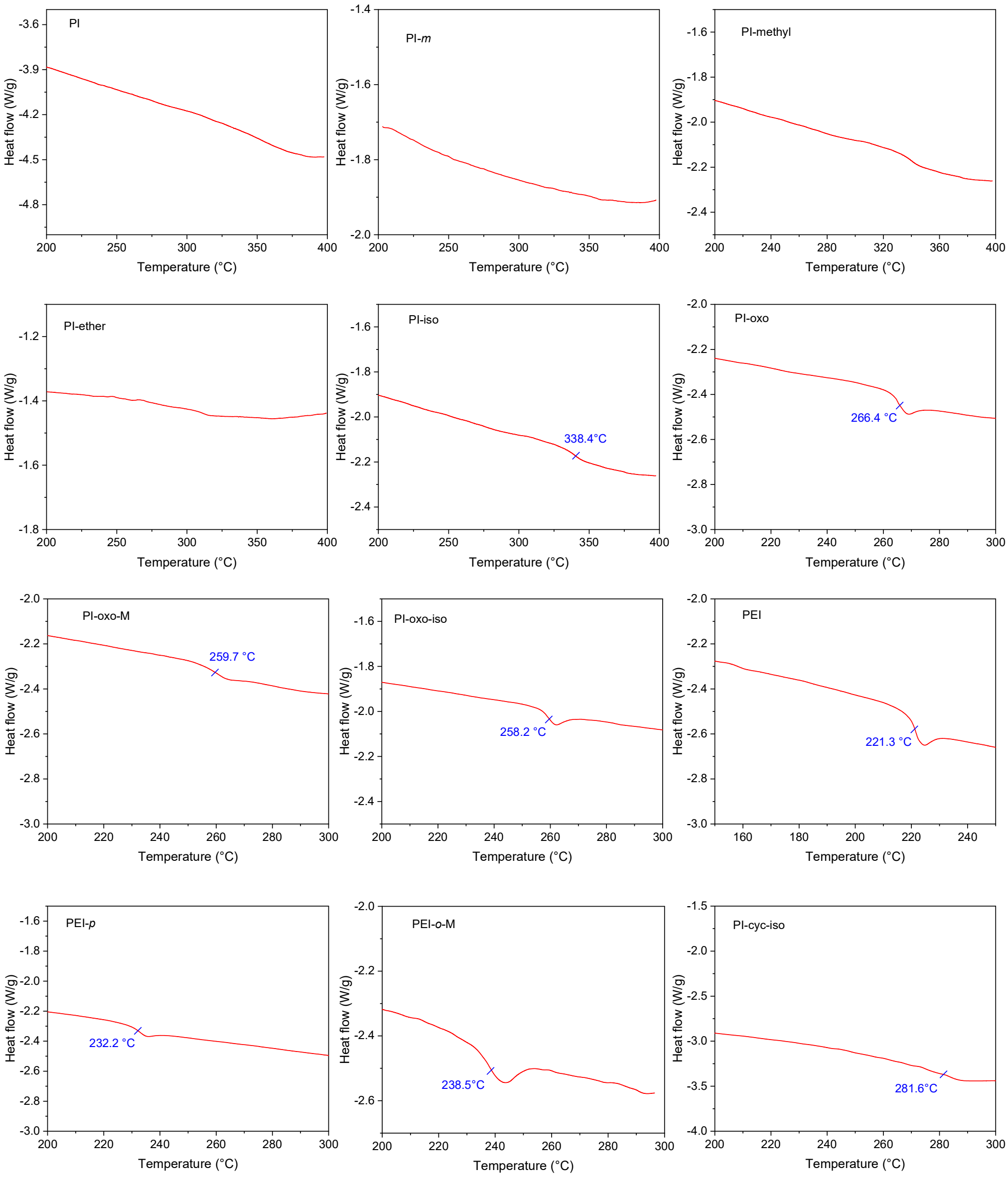

Supplementary Figure S17 Differential scanning calorimetric (DSC) curves of the experimental dielectric polymers in this work. For some PI-derived polymers that are difficult to accurately determine T_g in the DSC curve, we conducted additional dynamic mechanical analysis (DMA) tests to calibrate T_g (Supplementary Fig. S18).

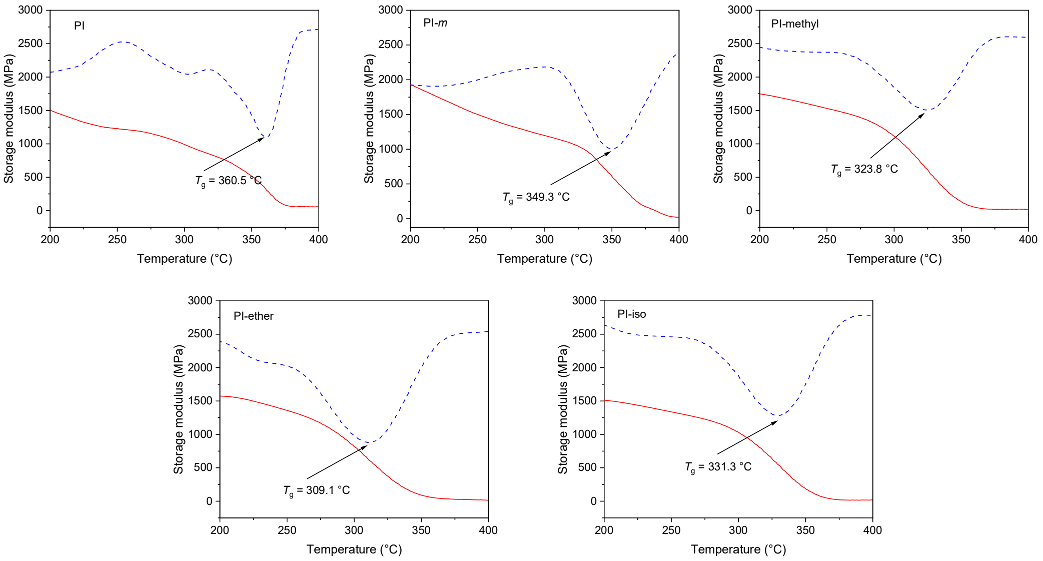

Supplementary Figure S18 Dynamic mechanical analysis (DMA) curves of some polymer dielectrics whose T_g cannot be accurately determined by DSC.

Supplementary Table S2. E_g from $(\alpha hv)^2$-hv plots and T_g from DSC/DMA of the experimental dielectric polymers in this work.

	E_g	T_g
PI	2.77	360.5 (DMA)
PI-*m*	2.75	349.3 (DMA)
PI-methyl	2.86	323.8 (DMA)
PI-ether	2.83	309.1 (DMA)
PI-iso	2.99	331.3 (DMA)
PI-oxo	3.3	266.4 (DSC)
PI-oxo-M	3.31	259.7 (DSC)
PI-oxo-iso	3.5	258.2 (DSC)
PEI	3.29	221.3 (DSC)
PEI-*p*	3.33	232.2 (DSC)
PEI-*o*-M	3.33	238.5 (DSC)
PI-cyc-iso	4.15	281.6 (DSC)

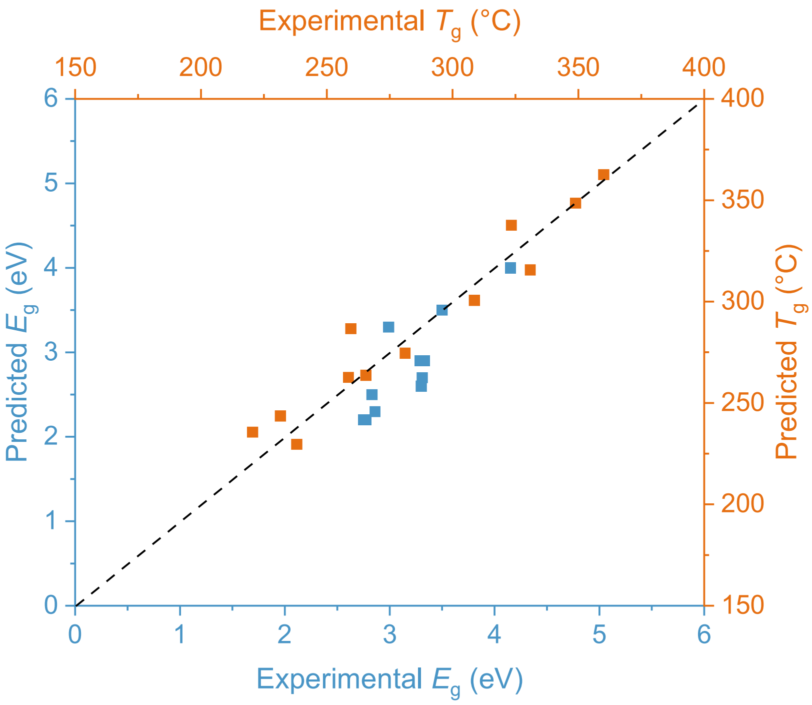

Supplementary Figure S19 Comparison of T_g and E_g of the selected 12 polymers between the experimental method and the theoretical calculation method.

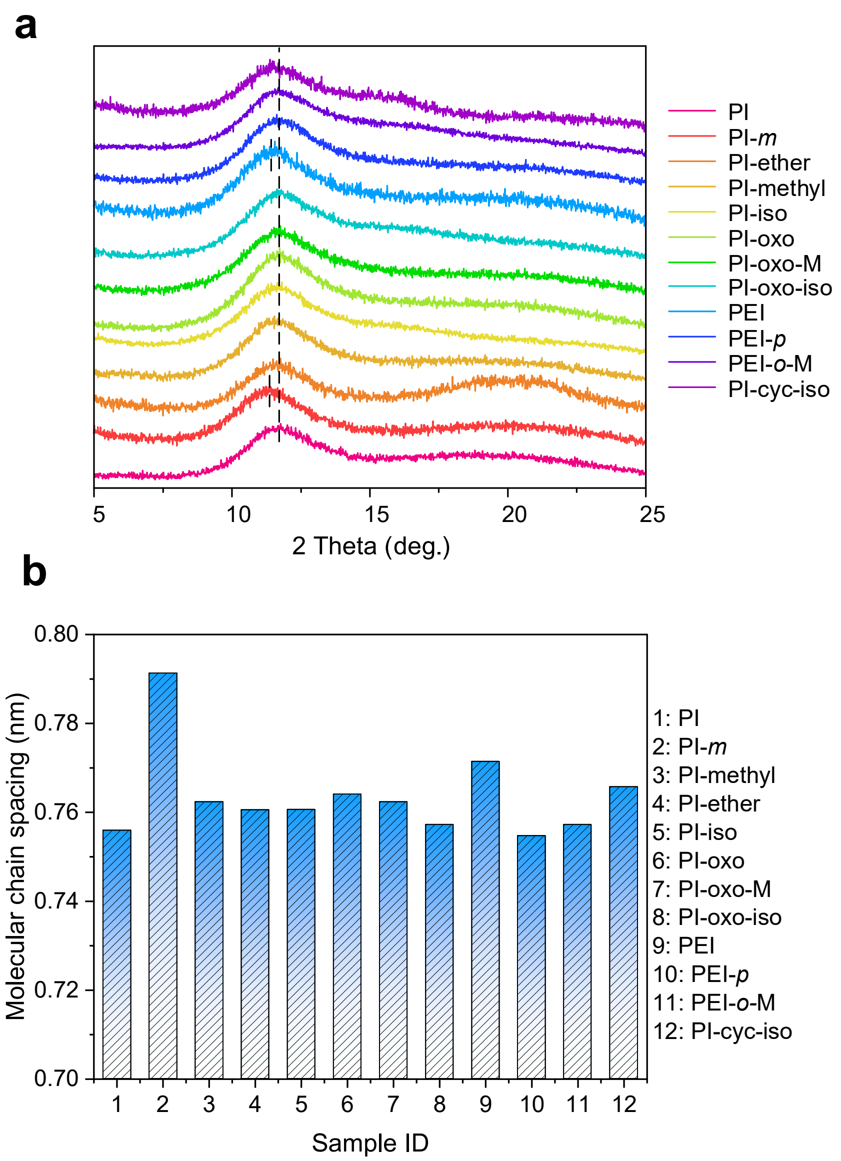

Supplementary Figure S20 (a) X-ray diffraction patterns of the experimental dielectric polymers in this work. (b) Molecular chain spacing obtained from XRD results calculated by Bragg equation. The Bragg equation is represented as $2d\sin\theta = n\lambda$, where d is the crystal plane spacing, θ is the angle between the incident X-ray and the corresponding crystal plane, λ Is the wavelength of X-ray, and n is the diffraction order. Obviously, the molecular chain spacing of the polymers containing *m*-benzene shows a significant increase, which is not conducive to the formation of preferred layer packing (PLP) and mixed layer packing (MLP), thus leading to the decrease of T_g. Except for *m*-benzene structure, other structural units have minor impact on the stacking of molecular chains, indicating that the rigidity of structural units is the main factor affecting the T_g of polymers.

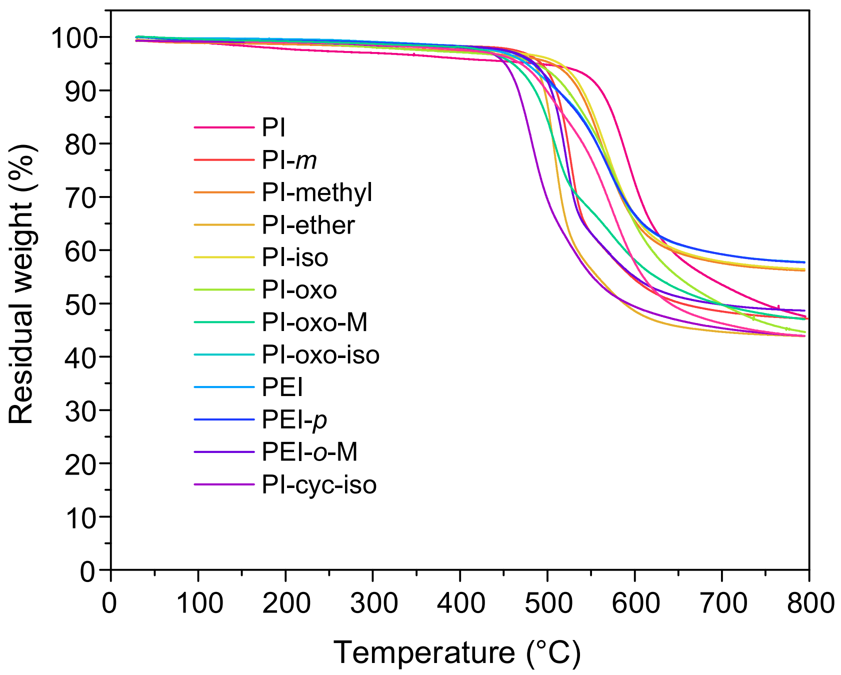

Supplementary Figure S21 Thermogravimetric analysis curves of the experimental dielectric polymers in this work. The results show that all polymers begin to decompose at temperatures above 400 °C, indicating excellent thermal stability. The final residual mass of the polymer is related to the carbon content, which can be attributed to the fact that the final residual material heated in nitrogen atmosphere is amorphous carbon.

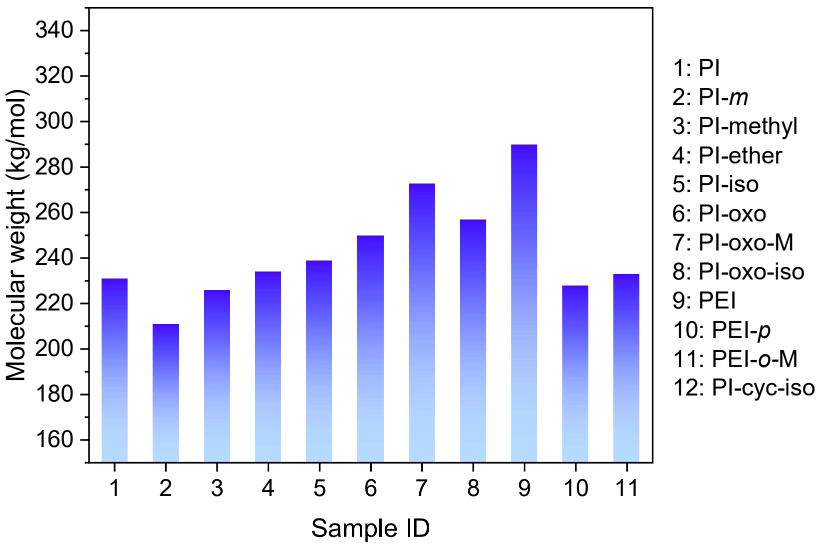

Supplementary Figure S22 Molecular weight from gel permeation chromatography (GPC) of the experimental dielectric polymers in this work. The results show that the M_w of 12 PI-derived polymers are distributed in the range of 200-300 kg/mol, indicating that the impact of this factor on the electrical and mechanical properties of polymers can be excluded.

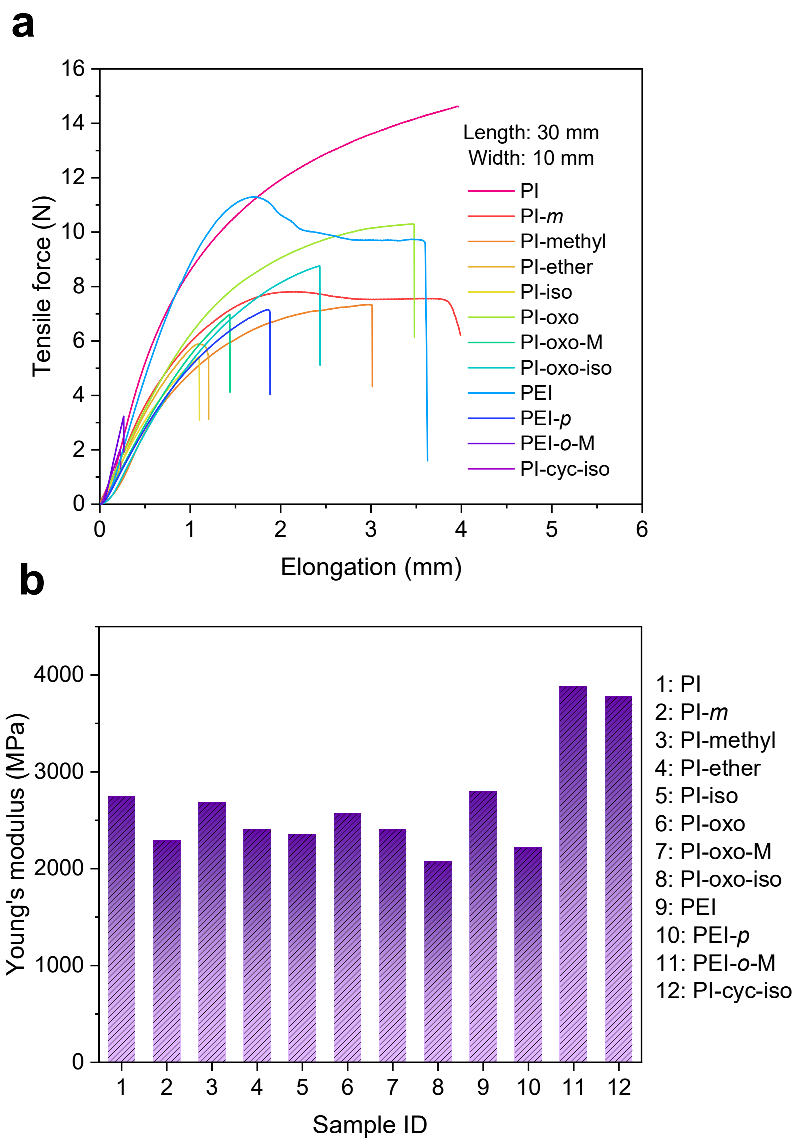

Supplementary Figure S23 (a) Stress strain curve and (b) Young's modulus of the experimental dielectric polymers in this work.

Section 2. High-field insulation and low-field dielectric properties

In order to further understand the conduction mechanism of charge in polymer, we used electrode-limited conduction mechanism and bulk phase-limited conduction mechanism to verify. The most common electrode-limited conduction mechanism in dielectrics at high temperatures is Schottky injection (also known as thermionic emission). The carrier is thermally excited to obtain enough energy to overcome the barrier at the interface, and the process of injection is called Schottky injection. Schottky injection current density can be expressed by,

$$J = AT^2 \exp\left(\frac{-q\left(\phi_B - \sqrt{qE / 4\pi\varepsilon_r\varepsilon_0}\right)}{k_B T} \right) \tag{1}$$

$$A = \frac{4\pi q k_B^2 m_0}{h^3} \tag{2}$$

where A is the Richardson constant, T is the absolute temperature, q is the charge of the carrier, $q\phi_B$ is the Schottky injection barrier, ε_r is the relative permittivity of the dielectric material, ε_0 is the vacuum dielectric constant, k_B is the Boltzmann constant, E is the electric field strength, m_0 is the free electron mass, and h is the Planck constant. Equation (1) can be equivalently transformed into,

$$\ln\left(\frac{J}{T^2}\right) = \left(\frac{\sqrt{q^3 / 4\pi\varepsilon_r\varepsilon_0}}{k_B T}\right)\sqrt{E} + \ln(A) - \left(\frac{q\phi_B}{k_B T}\right) \tag{3}$$

Therefore, for the Schottky injection model, the relationship between $\ln(J/T^2)$ and $E^{1/2}$ is linear. The linear fitting results of $\ln(J)$ on $E^{1/2}$ can be used to determine whether the Schottky injection is the dominant conduction mechanism. However, it should be noted that the fitting slope is related to the dielectric constant of the material. Even if the fitting degree is high, if the dielectric constant derived from the slope does not match the experimental dielectric constant, it is not in the mechanism of Schottky injection. The intercept of the fitting line of the Schottky diagram is related to the barrier height. At the same temperature, the smaller intercept means the higher Schottky injection barrier.

The conduction mechanism of the bulk phase limitation is Poole-Frenkel (P-F) emission and solid-state hopping conductance. P-F emission refers to the process that electrons captured by traps in the dielectric material are heated and excited into the conduction band to form current. By applying an electric field on the dielectric, the potential energy of electrons can be reduced, thus increasing the probability of electrons being heated and excited. Therefore, P-F emission usually appears at high temperature and high electric field. P-F emission current density can be given as,

$$J = q\mu N_C E \exp\left(\frac{-q\left(\phi_T - \sqrt{qE / \pi\varepsilon_r\varepsilon_0}\right)}{k_B T}\right) \tag{4}$$

where μ is the carrier migration rate, N_C is the state density of conduction band, $q\phi_T$ is the trap level (*i.e.* P-F emission activation energy), and other parameters have the same meanings as those in the Schottky injection current density formula. Equation (4) can be equivalently transformed into,

$$\ln\left(J / E\right) = \frac{\sqrt{q^3 / \pi\varepsilon_r\varepsilon_0}}{k_B T}\sqrt{E} + \ln\left(q\mu N_C\right) - \frac{q\phi_T}{k_B T} \tag{5}$$

For P-F emission, $\ln(J/T^2)$ is linear with $E^{1/2}$. The linear fitting results of $\ln(J/T^2)$ on $E^{1/2}$ can be used to determine whether the P-F emission is the dominant conduction mechanism. The determination rule is similar to the Schottky injection. The slope of the fitting line is related to the dielectric constant of the dielectric, and the intercept of the fitting line is related to the trap energy level.

P-F emission is similar to Schottky injection, while hopping conduction is similar to tunneling effect. In the hopping conduction, carriers trapped by traps cannot obtain enough energy to be excited into the conduction band, but they can still "jump" between different traps through tunneling effect. The current density generated by hopping conductance can be expressed as,

$$J = 2nq\lambda v \exp\left(-\frac{E_a}{k_B T}\right)\sinh\left(\frac{\lambda qE}{2k_B T}\right) \tag{6}$$

where n is the carrier density, q is the electric charge of the carriers, λ is the average hopping distance, v Is the attempt-to-escape frequency, E_a is the activation energy of

hopping conductance, k_B is the Boltzmann constant, T is the absolute temperature, and E is the electric field strength. The hyperbolic sine function can be approximated to an exponential function at high electric field. Thus, the Equation (6) can be transformed into,

$$J = 2nq\lambda v \exp\left(\frac{\lambda qE}{2k_BT} - \frac{E_a}{k_BT}\right) \tag{7}$$

From the above formula, for the leakage current J generated by the hopping conductance, the relationship between ln$J$ and E is linear at high electric field. Generally, when the temperature is fixed, the hyperbolic sine function can be given as,

$$J = \alpha \sinh(\beta E) \tag{8}$$

$$\alpha = 2nq\lambda v \exp\left(-\frac{E_a}{k_BT}\right) \tag{9}$$

$$\beta = \frac{\lambda q}{2k_BT} \tag{10}$$

The leakage current density measured under different electric field intensity is fitted to determine whether the dominant conduction mechanism is the hopping conduction. In the formula, the fitting parameter β is a constant related to the hopping distance, which can be used to calculate the hopping distance.

For the sake of a brief discussion of the TSDC theory, only the electrons injected from the electrode will be considered in the following. In case of a continuous trap level distribution with the distribution function, which is spatially uniform deep into the film with a distance l, the induced current in the external circuit during TSC measurement can be calculated as follows[3],

$$J(T) = \frac{el^2}{2d}\int_{E_v}^{E_c} f_0(E)N_t(E)e_n(E,T)e^{-\frac{1}{\beta}\int_{T_0}^{T} e_n(E,T)\mathrm{d}T}\,\mathrm{d}E \tag{11}$$

where e is electronic charge quantity，f_0 is the initial occupancy of a trap level and is a constant, E is the trap energy (trap depth). $e_n(E,T)=v\exp(-E_t/kT)$ is the emission rate of electrons at trap level E and temperature T. k is the Boltzmann constant, d is the thickness of the film and β is the heating rate. v is commonly called the frequency

factor or attempt-to-escape frequency. The usual interpretation of v is that it represents the number of times per second a bound electron interacts with the lattice phonons. The normal value expected for v is therefore the lattice vibration frequency[3], typically 10^{12}–10^{14} s^{-1}. In this paper, v was assumed to be 10^{12} s^{-1} as suggested by Mott[4]. From Supplementary Equation 11, the plot of charge density versus temperature can be converted into a diagram of trap density versus energy level[5].

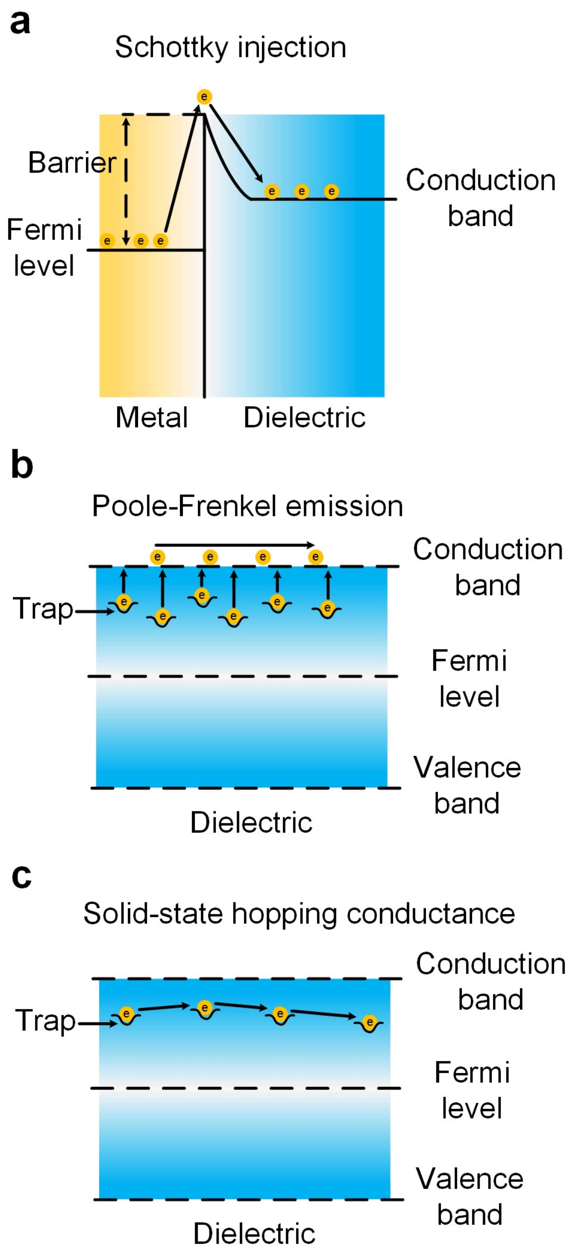

Supplementary Figure S24 Energy band diagram of (a) Schottky injection, (b) Poole-Frenkel emission and (c) solid-state hopping conductivity.

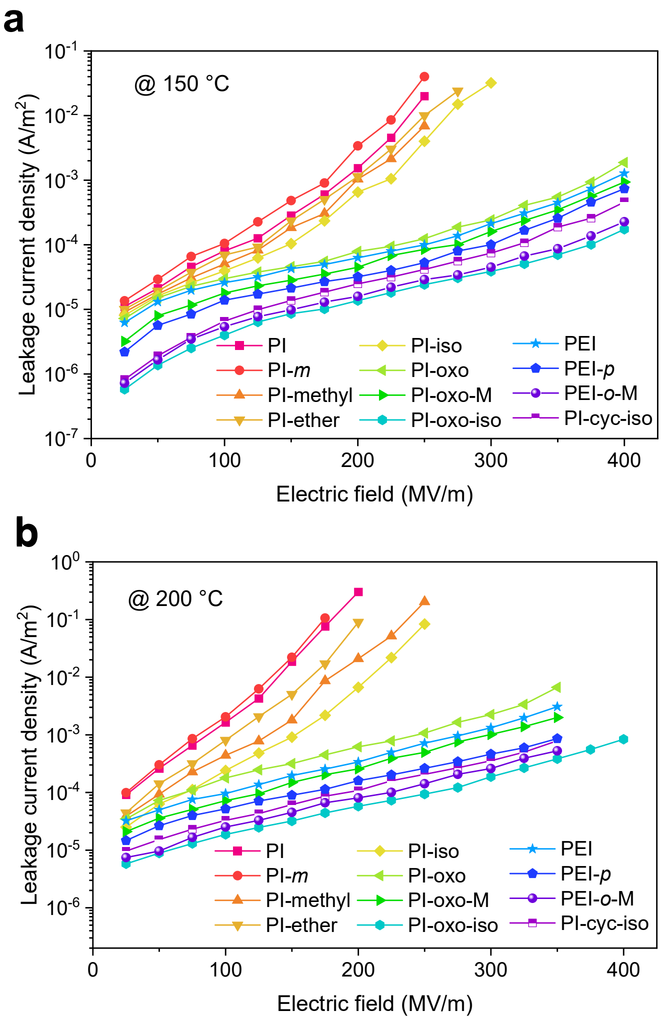

Supplementary Figure S25 Field-dependent leakage current density of the various dielectric polymers at (a) 150 °C and (b) 200 °C.

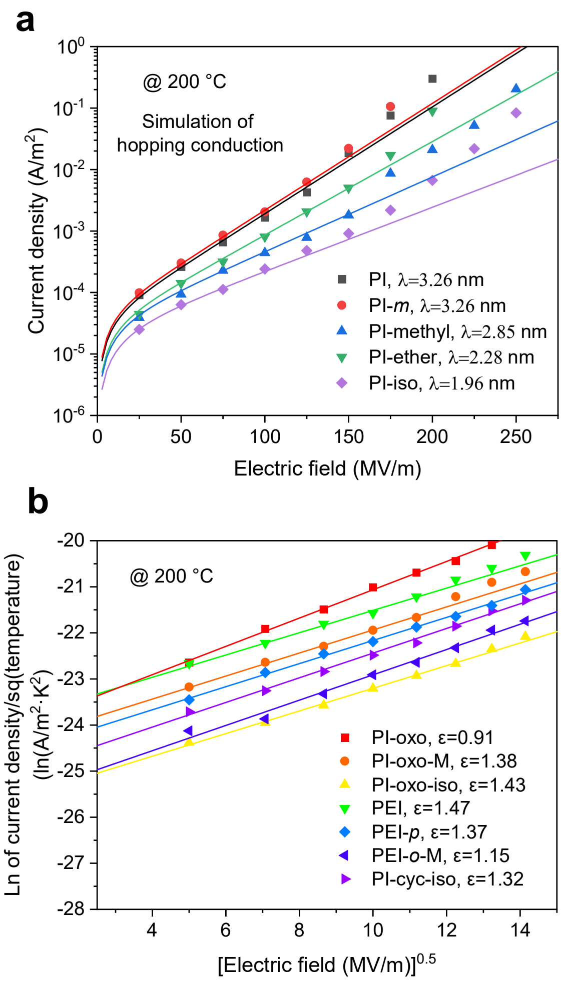

Supplementary Figure S26 Current density of the PI-derived polymer synthesized from (a) pyromellitic dianhydride and (b) long-chain dianhydride monomers as a function of electric field at 200 °C. The fitting dielectric constant is significantly lower than that measured in the experiment, indicating that Schottky injection and Poole-Frenkel emission is not the dominant conductance mechanism.

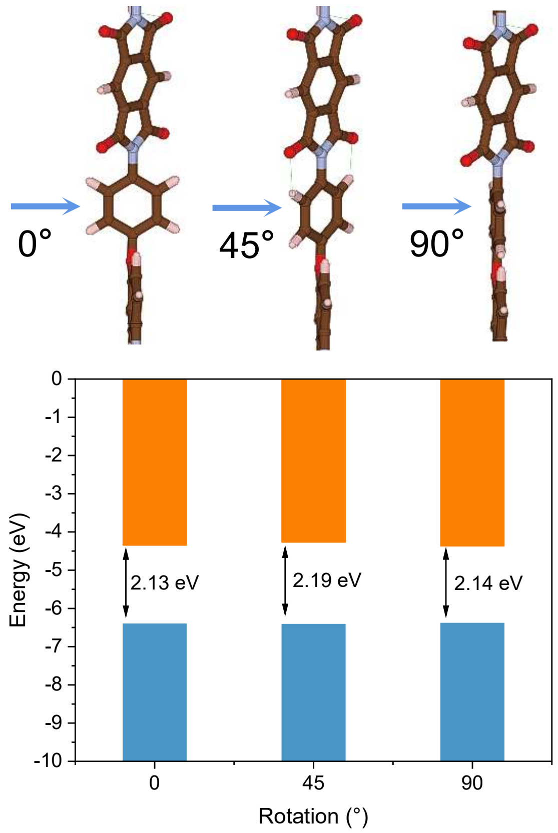

Supplementary Figure S27 Conduction band and valence band of PI with different dihedral angle between neighboring planes of conjugation from DFT methods.

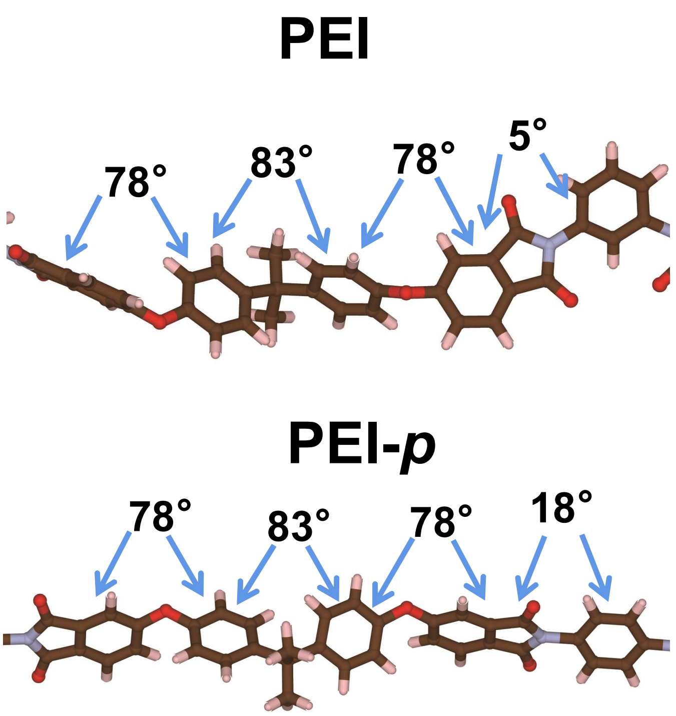

Supplementary Figure S28 Schematic diagram of five polymer chains at the lowest energy.

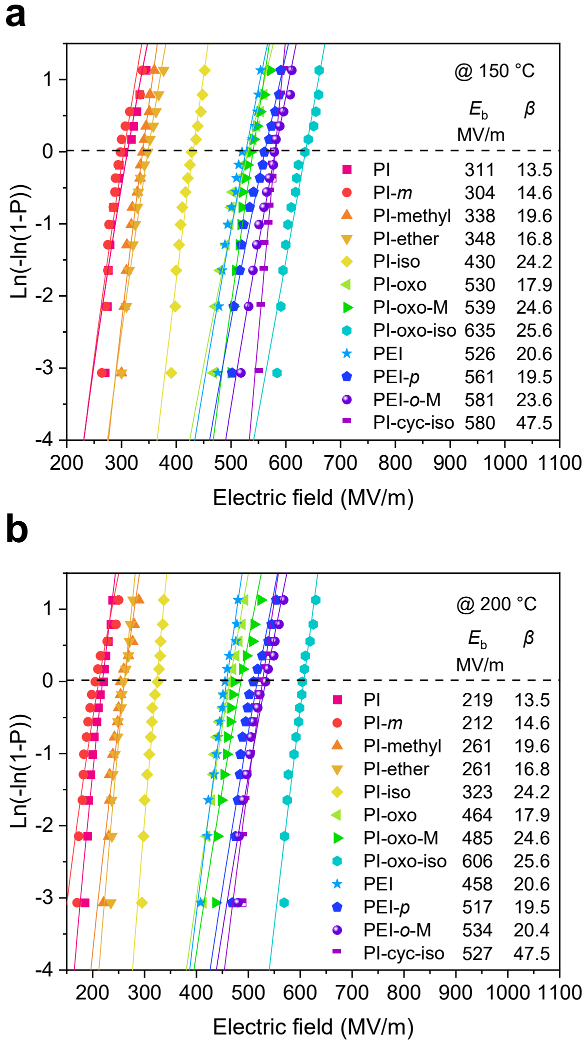

Supplementary Figure S29 Weibull distribution analysis of the breakdown strengths of the various dielectric polymers at (a) 150 °C and (b) 200 °C. In the breakdown strength test, we judge the voltage at the beginning of burr on the I-V curve as the breakdown point.

Supplementary Table S3. Comparison of θ, leakage current density, hopping distance, breakdown strength and E_g of PI-derived polymers with hopping conduction. Hopping distance is obtained from leakage current density of the polymer conforming to hopping conduction (Equation (6)), and a shorter hopping distance corresponds to a deeper trap depth[6].

	θ (°)	Leakage current density (A/m^2) at 200 °C under 300 MV/m	Hopping distance (nm)	Breakdown strength (MV/m)	E_g
PI	7.5	/	/	219	2.77
PI-m	9	/	/	212	2.75
PI-methyl	22.5	/	/	261	2.86
PI-ether	10	/	/	261	2.83
PI-iso	55.3	/	/	323	2.99
PI-oxo	46.5	2.2×10^{-3}	1.1	464	3.3
PI-oxo-M	61.5	1.0×10^{-3}	0.94	485	3.31
PI-oxo-iso	81.3	1.9×10^{-4}	0.89	606	3.5
PEI	60.5	1.3×10^{-3}	1.01	458	3.29
PEI-*p*	64.3	4.6×10^{-4}	0.94	517	3.33
PEI-*o*-M	75.3	2.6×10^{-4}	0.9	534	3.33
PI-cyc-iso	/	3.54×10^{-4}	0.91	527	4.15

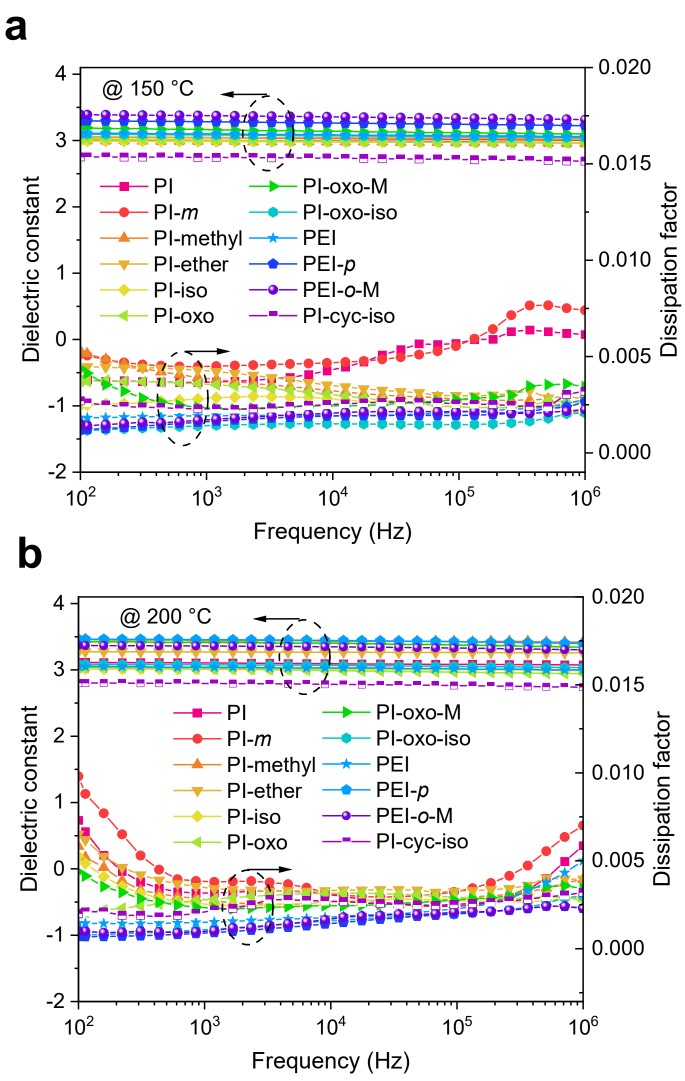

Supplementary Figure S30 Frequency-dependent dielectric properties of the various dielectric polymers at (a) 150 °C and (b) 200 °C.

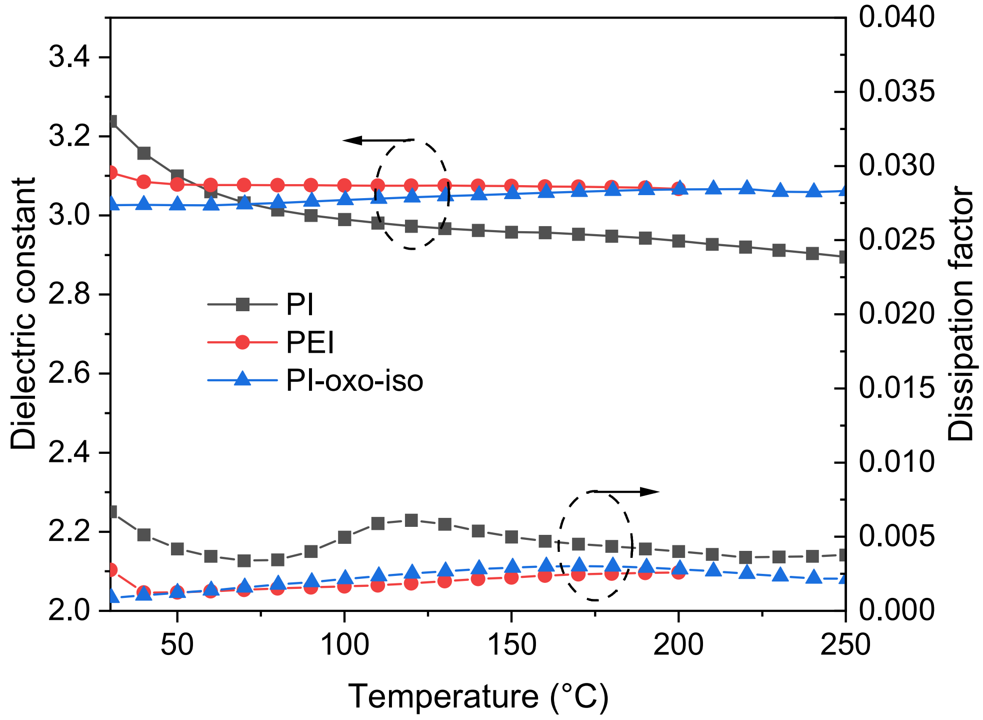

Supplementary Figure S31 Temperature-dependent dielectric properties of the PI, PEI, and PI-oxo-iso at 1000 Hz.

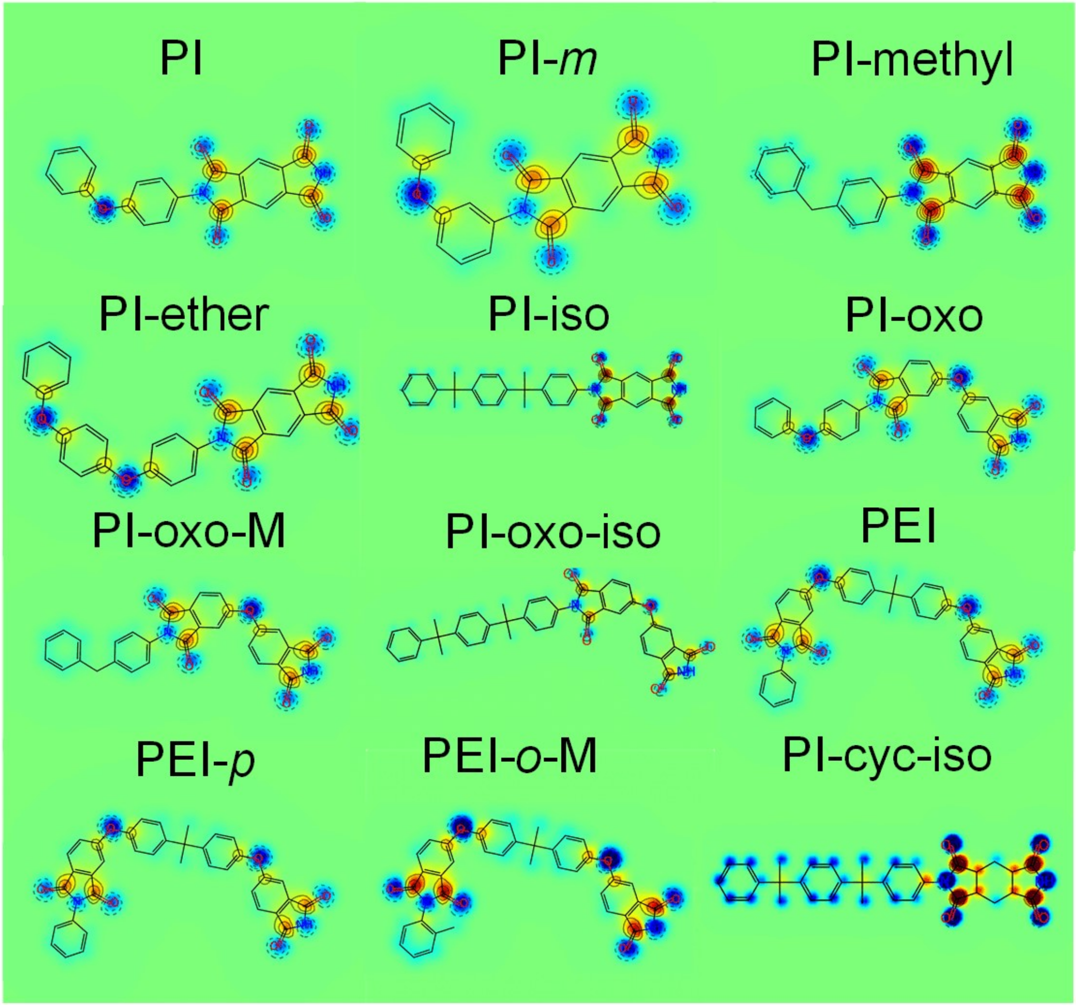

Supplementary Figure S32 The Gasteiger charges distribution of the experimental dielectric polymers in this work. The Gasteiger charges distribution implies that the negative charge has a high concentration near the oxygen atom, while the carbon atom connected to it has a corresponding high concentration of positive charge. Therefore, the oxygen atom with negative potential in the diamine monomer may also become a bridge for electron transfer between adjacent benzene rings, resulting in low trap levels.

Section 3. High temperature capacitance performance

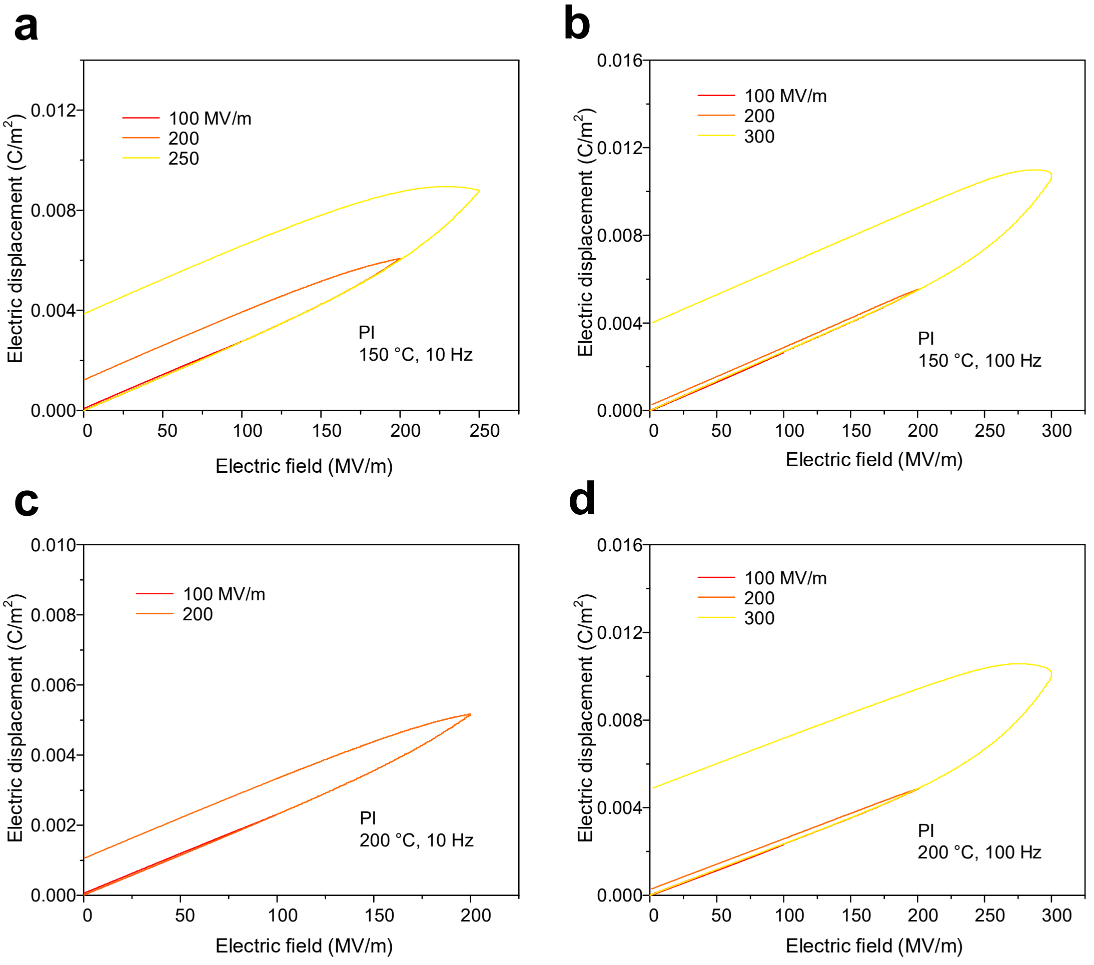

Supplementary Figure S33 Unipolar *D-E* loops of PI measured at (a) 150 °C with 10 Hz, (b) 150 °C with 100 Hz, (c) 200 °C with 10 Hz, and (d) 200 °C with 100 Hz.

.

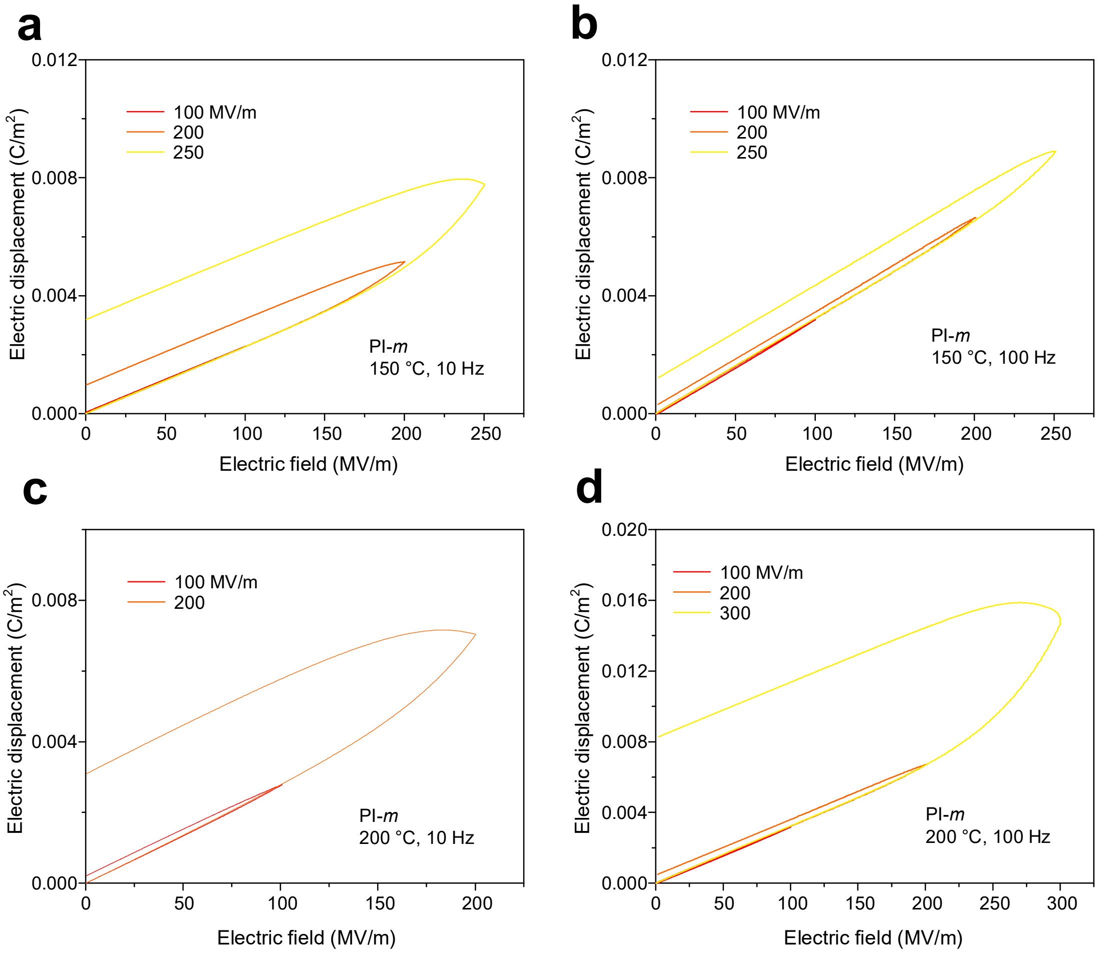

Supplementary Figure S34 Unipolar *D-E* loops of PI-*m* measured at (a) 150°C with 10 Hz, (b) 150°C with 100 Hz, (c) 200°C with 10 Hz, and (d) 200°C with 100 Hz.

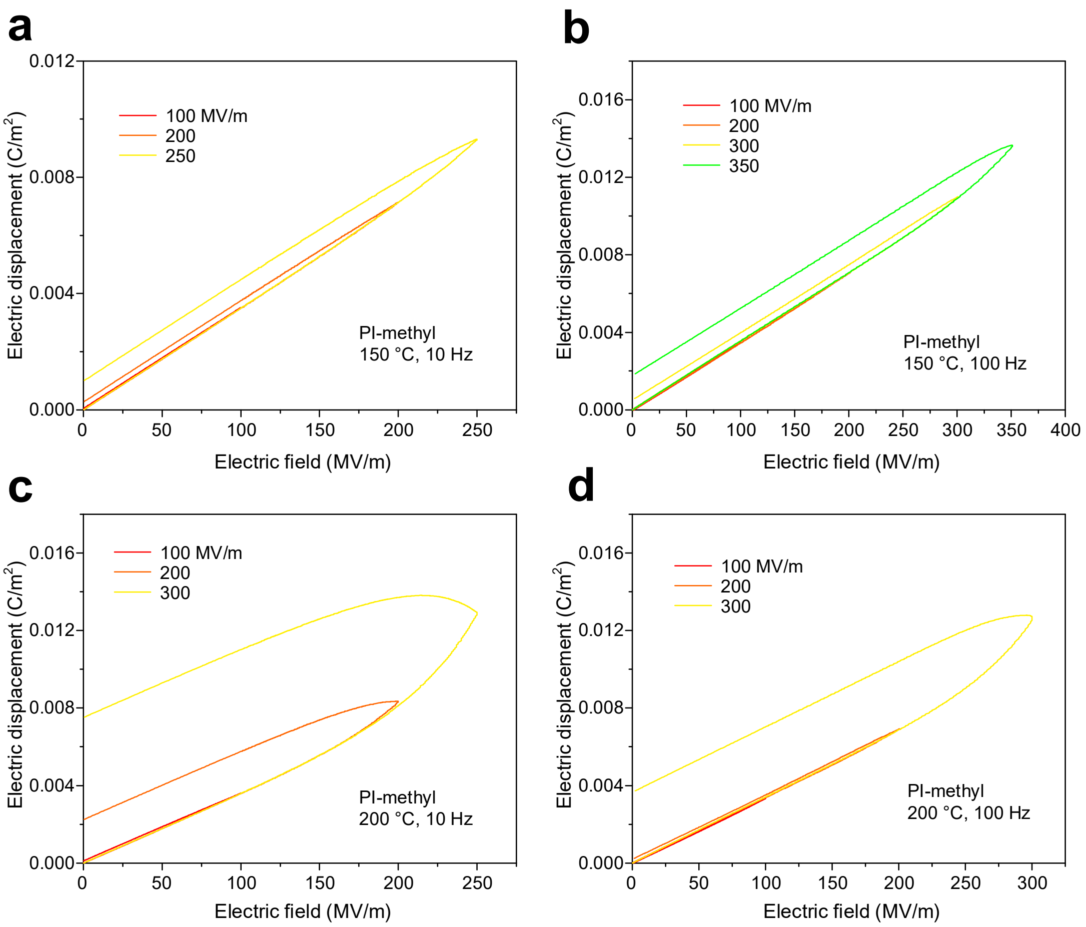

Supplementary Figure S35 Unipolar *D-E* loops of PI-methyl measured at (a) 150 °C with 10 Hz, (b) 150 °C with 100 Hz, (c) 200 °C with 10 Hz, and (d) 200 °C with 100 Hz.

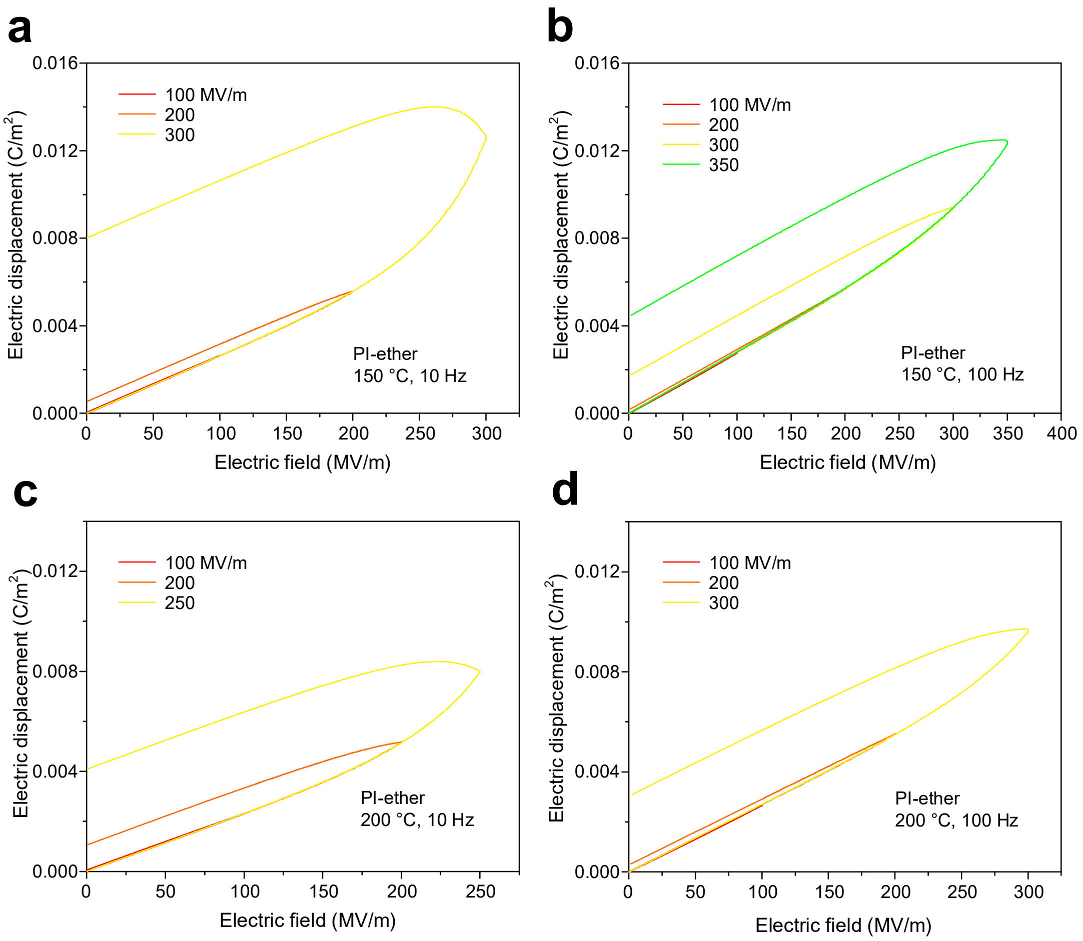

Supplementary Figure S36 Unipolar *D-E* loops of PI-ether measured at (a) 150 °C with 10 Hz, (b) 150 °C with 100 Hz, (c) 200 °C with 10 Hz, and (d) 200 °C with 100 Hz.

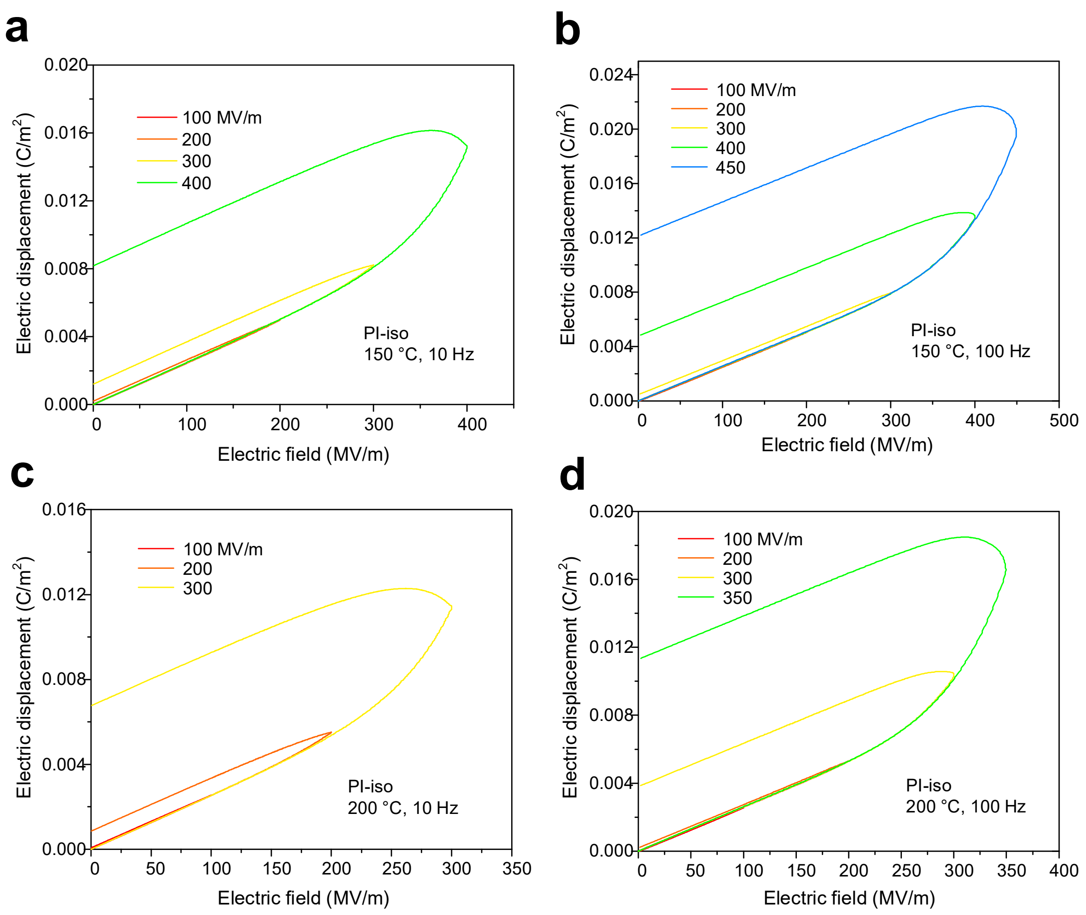

Supplementary Figure S37 Unipolar *D-E* loops of PI-iso measured at (a) 150 °C with 10 Hz, (b) 150 °C with 100 Hz, (c) 200 °C with 10 Hz, and (d) 200 °C with 100 Hz.

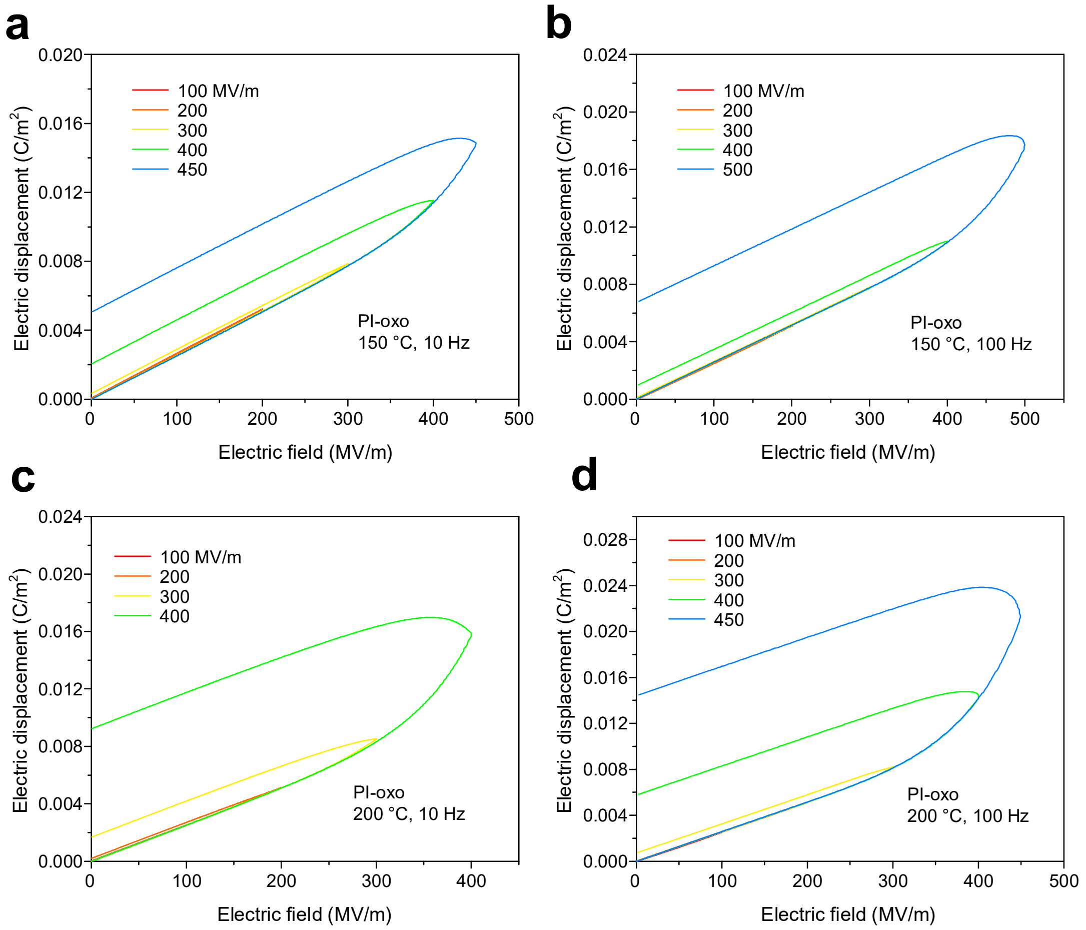

Supplementary Figure S38 Unipolar *D-E* loops of PI-oxo measured at (a) 150 °C with 10 Hz, (b) 150 °C with 100 Hz, (c) 200 °C with 10 Hz, and (d) 200 °C with 100 Hz.

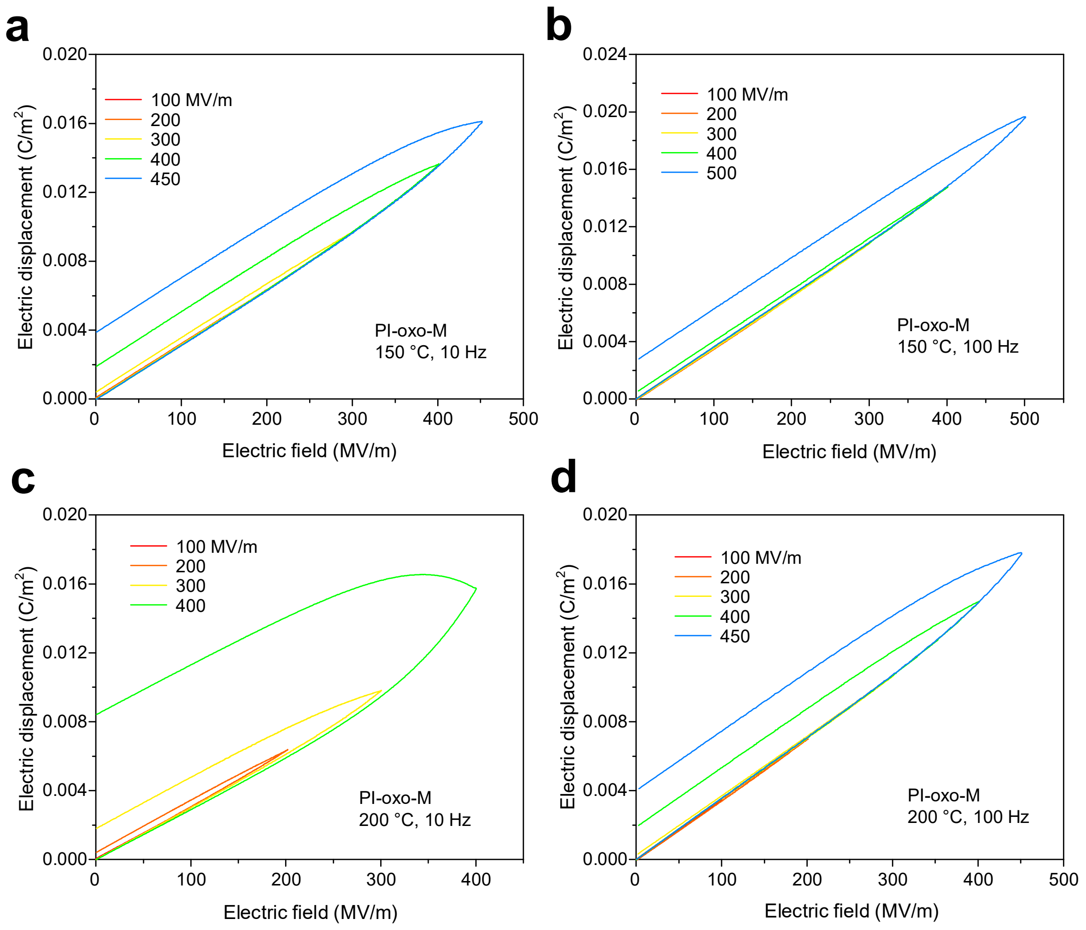

Supplementary Figure S39 Unipolar *D-E* loops of PI-oxo-M measured at (a) 150 °C with 10 Hz, (b) 150 °C with 100 Hz, (c) 200 °C with 10 Hz, and (d) 200 °C with 100 Hz.

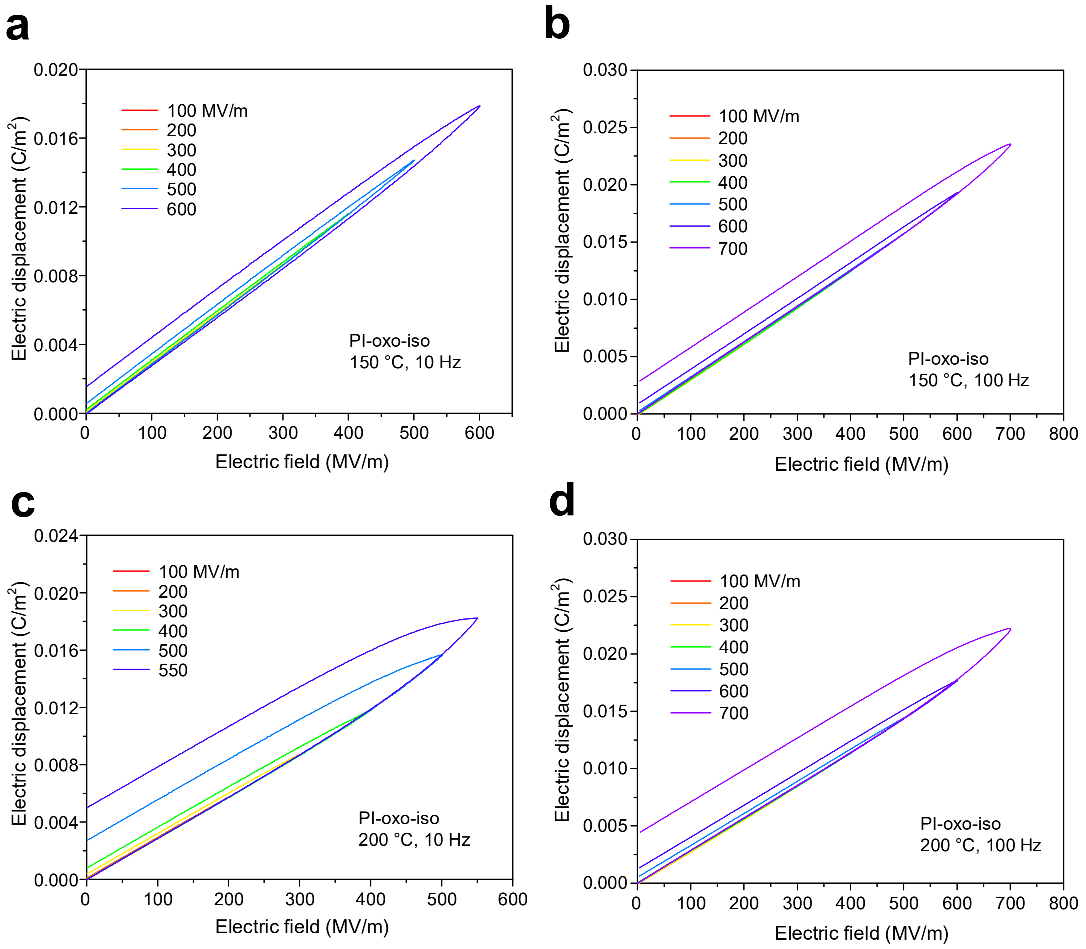

Supplementary Figure S40 Unipolar *D-E* loops of PI-oxo-iso measured at (a) 150 °C with 10 Hz, (b) 150 °C with 100 Hz, (c) 200 °C with 10 Hz, and (d) 200 °C with 100 Hz.

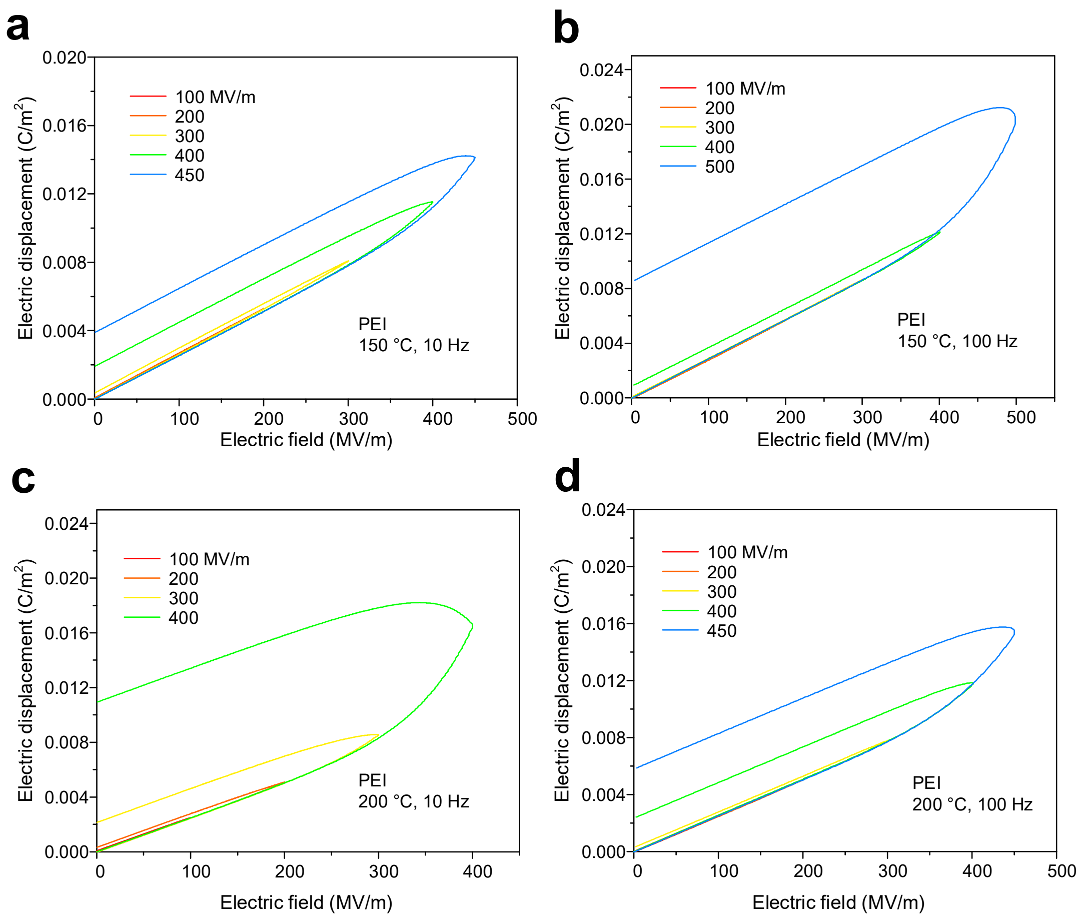

Supplementary Figure S41 Unipolar *D-E* loops of PEI measured at (a) 150 °C with 10 Hz, (b) 150 °C with 100 Hz, (c) 200 °C with 10 Hz, and (d) 200 °C with 100 Hz.

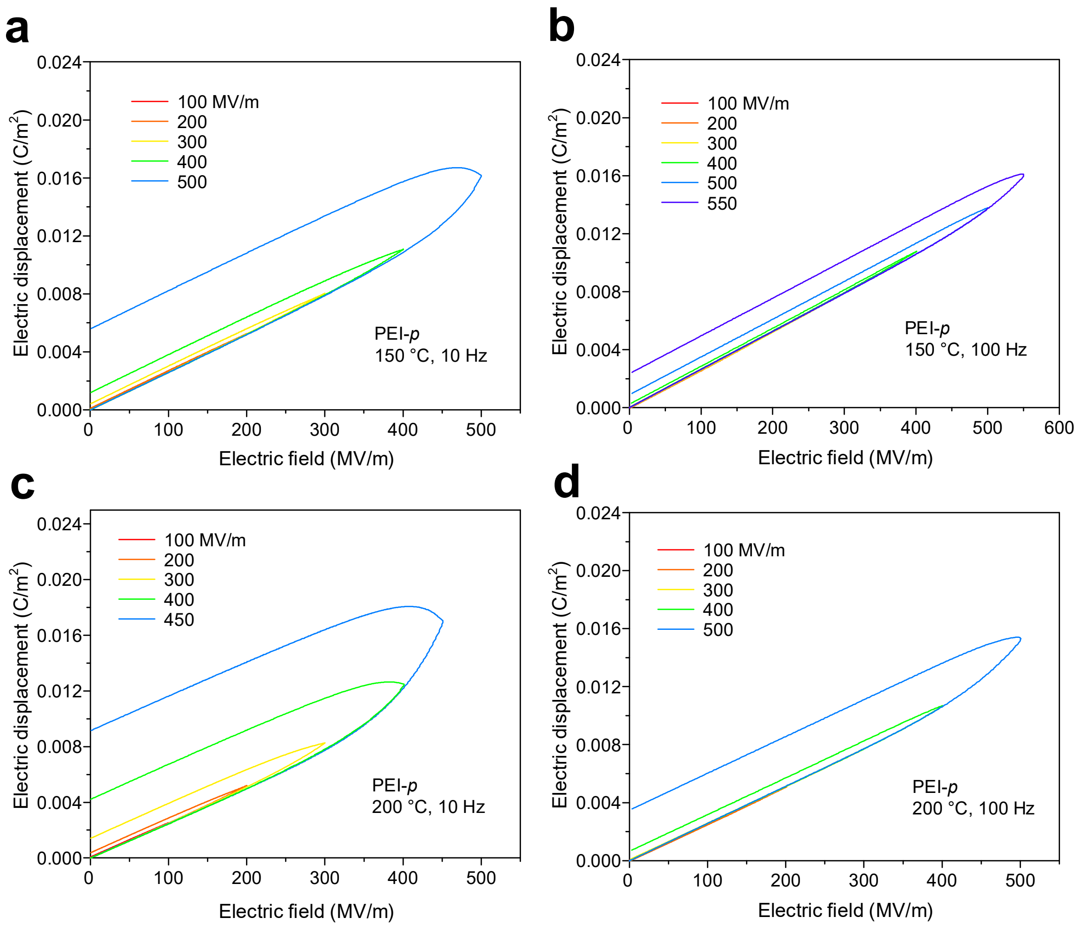

Supplementary Figure S42 Unipolar *D-E* loops of PEI-*p* measured at (a) 150 °C with 10 Hz, (b) 150 °C with 100 Hz, (c) 200 °C with 10 Hz, and (d) 200 °C with 100 Hz.

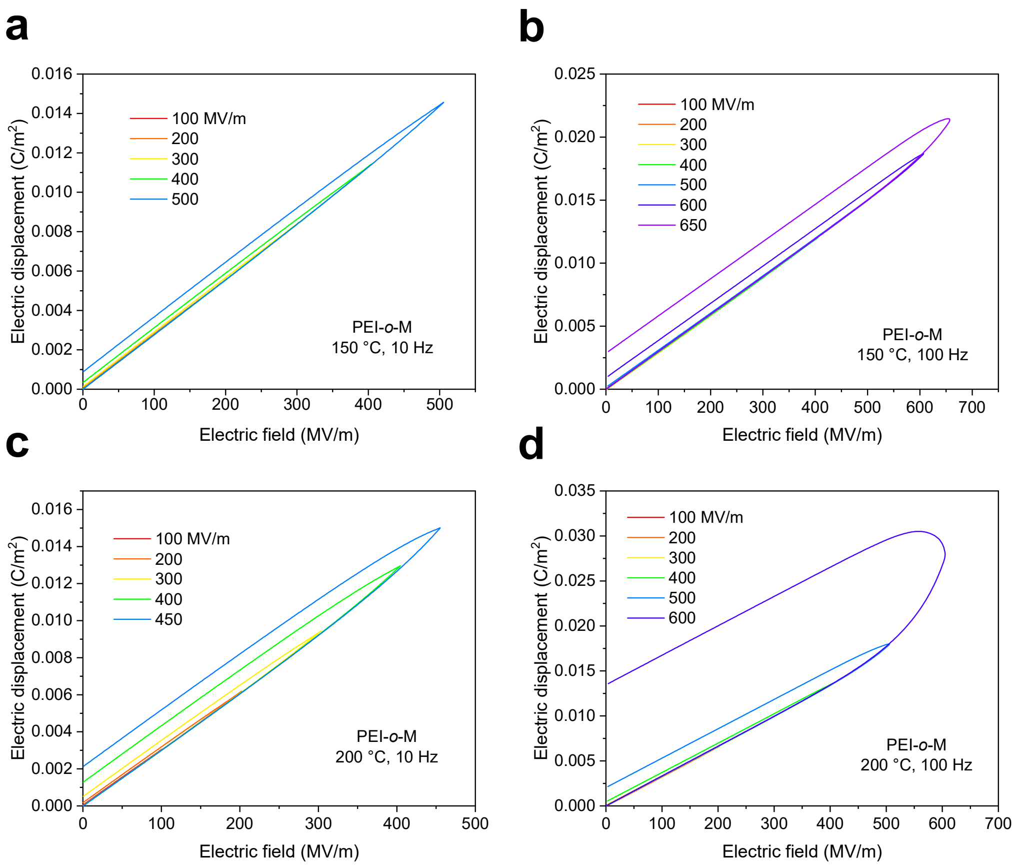

Supplementary Figure S43 Unipolar *D-E* loops of PEI-*o*-M measured at (a) 150 °C with 10 Hz, (b) 150 °C with 100 Hz, (c) 200 °C with 10 Hz, and (d) 200 °C with 100 Hz.

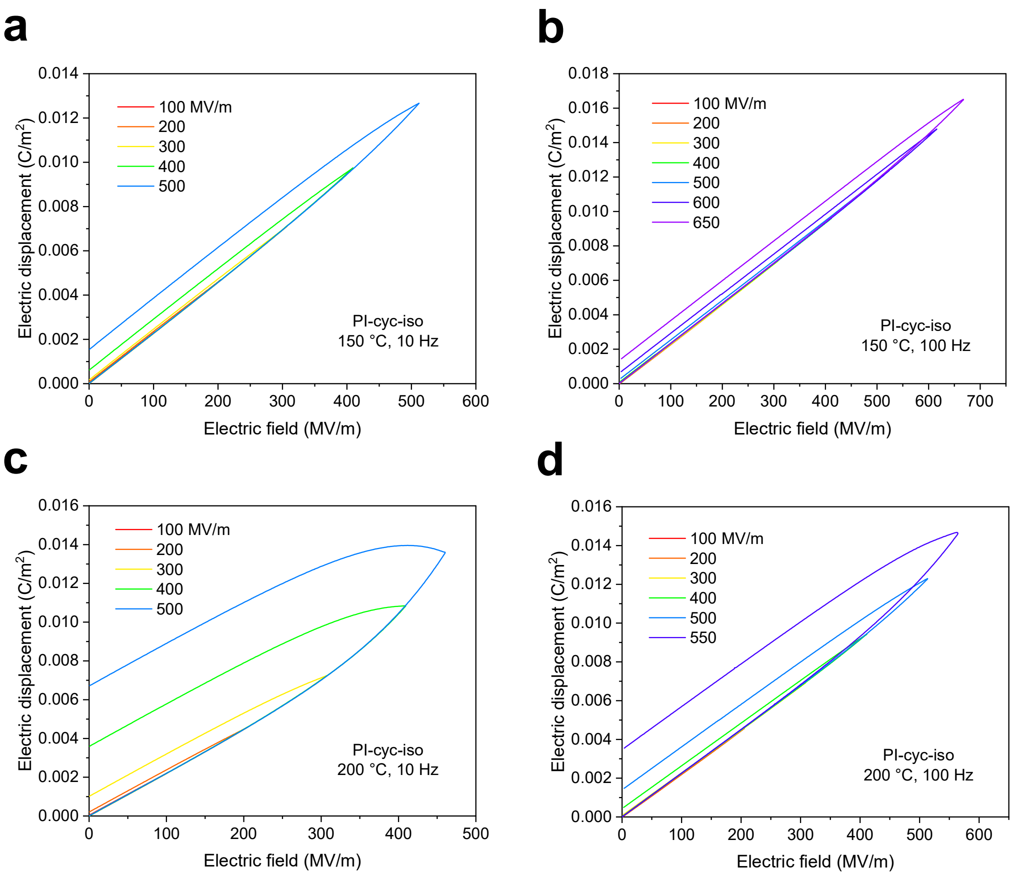

Supplementary Figure S44 Unipolar *D-E* loops of PI-cyc-iso measured at (a) 150 °C with 10 Hz, (b) 150 °C with 100 Hz, (c) 200 °C with 10 Hz, and (d) 200 °C with 100 Hz.

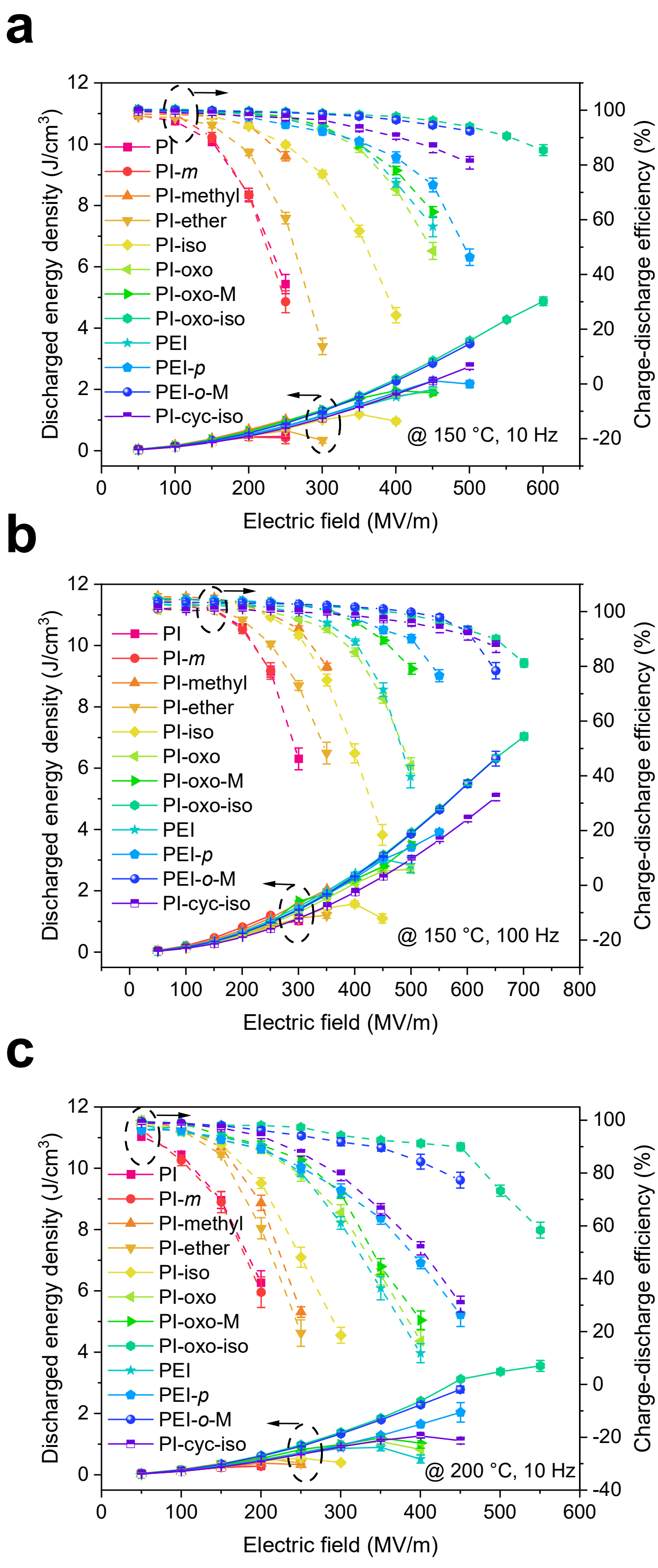

Supplementary Figure S45 Field-dependent charge-discharge efficiency and discharged energy density of the various dielectric polymers at (a) 150 °C with 10 Hz, (b) 150 °C with 100 Hz, and (c) 200 °C with 10 Hz. The average values and max–min error bars of the results were obtained from six parallel samples.

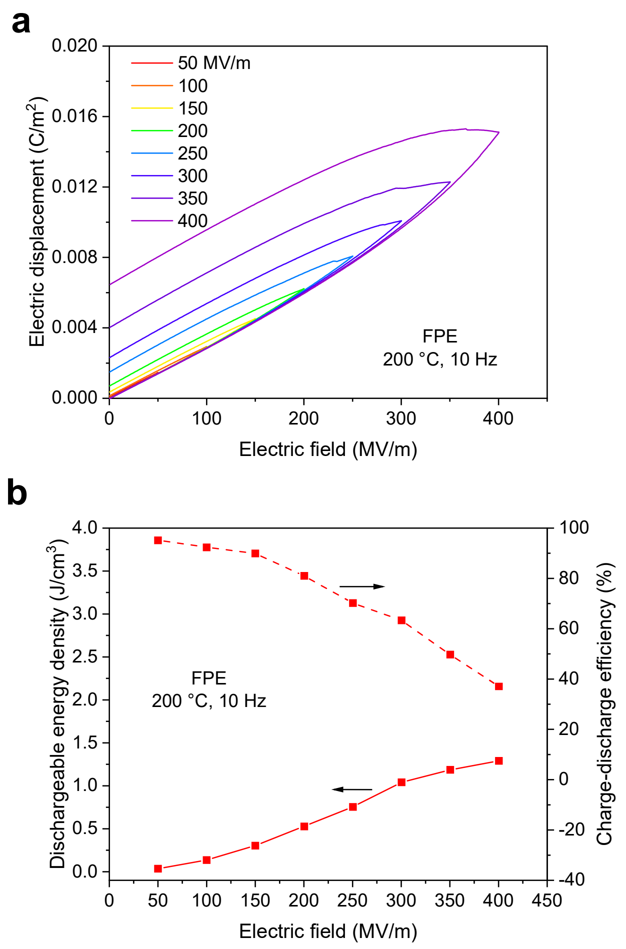

Supplementary Figure S46 (a) Unipolar *D-E* loops of FPE measured at 200 °C with 10 Hz. (b) Field-dependent charge-discharge efficiency and discharged energy density of FPE at 200 °C with 10 Hz.

Supplementary Table S4. The maximum discharged energy density and electric field strength of the most advanced polymer based high-temperature dielectrics reported at above 90% efficiency[7-16].

Materials	Discharged energy density (J/cm^3)	Electric field strength (MV/m)	Test frequency (Hz)	Ref.
PEI	0.472	250	10	This work
FPE	0.3	150	10	This work
PEI/PCBM	3	430	10	8
c-BCB/BNNS	0.56	200	10	7
PEI coated BN	1.18	300	10	14
PEI coated Al_2O_3	208	420	10	13
Al_2O_3-PI-Al_2O_3-PI-Al_2O_3	1.56	300	100	11
Al_2O_3-BT@SiO_2/PI-Al_2O_3	1.8	300	100	15
PI/BNNS	1.1	250	100	10
PEIs-PI-PEIs	2	300	100	9
o-POFNB	2.1	400	100	12
F-PI/PCBM	4.39	580	100	16
PI-oxo-iso	3.1	450	10	This work
PI-oxo-iso	5.324	600	100	This work

Section 4. Film quality

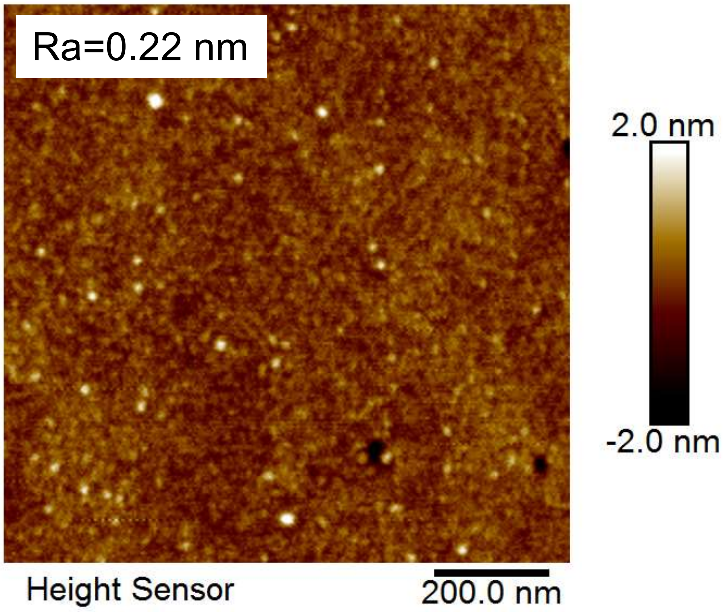

Supplementary Figure S47 Topography of the PI-oxo-iso film from atomic force microscope. The upper left corner shows that the roughness of the film is 0.22 nm. Such a small roughness proves that the surface of the material we prepared is extremely flat, which is very suitable for capacitor applications.

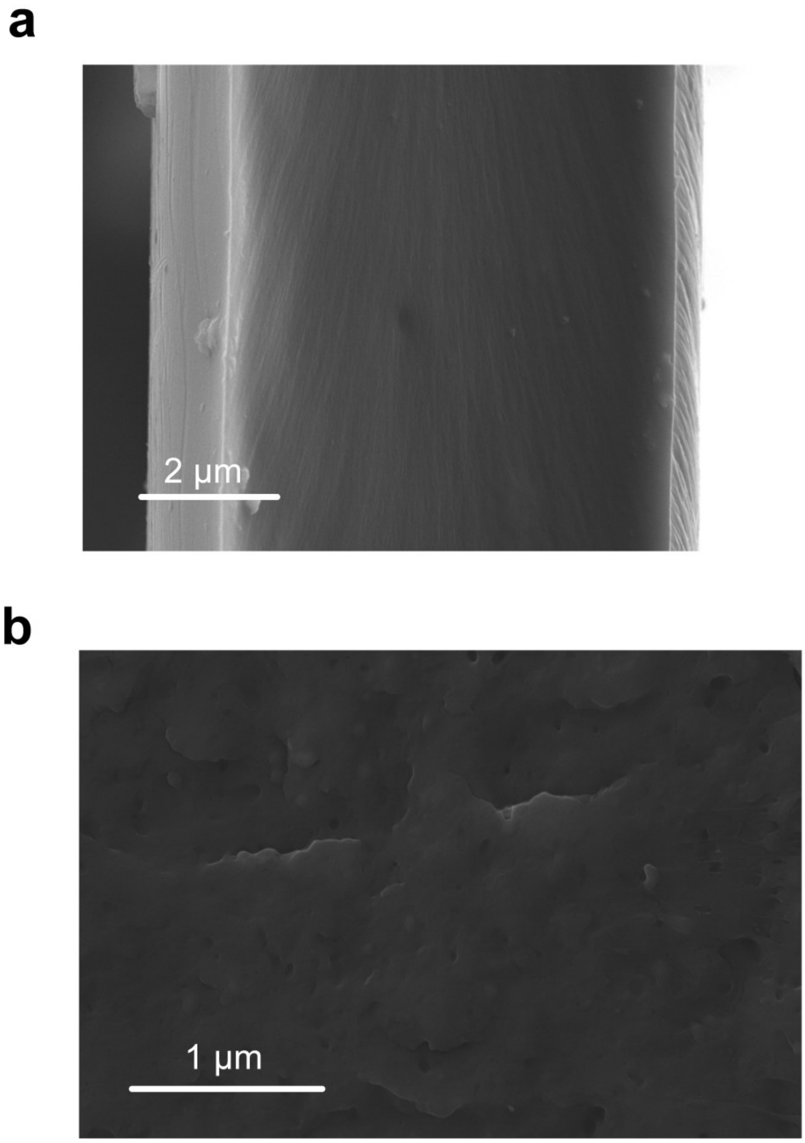

Supplementary Figure S48 Cross-sectional SEM images of PI-oxo-iso. Scale bar, (a) 2 µm, (b) 1 µm.

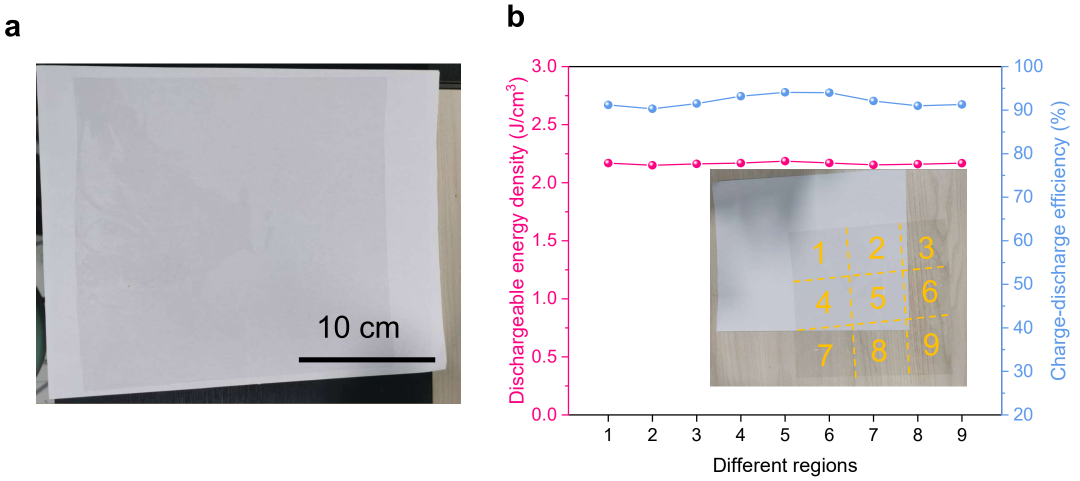

Supplementary Figure S49 (a) Photograph of a PI-oxo-iso film of 20 × 20 cm^2 area. An A4 paper sheet is stacked underneath the film as a reference. (b) Dischargeable energy density and charge-discharge efficiency tested at different regions (as marked in the inset photograph) of the large PI-oxo-iso film under 400 MV/m at 200 °C with 10 Hz.

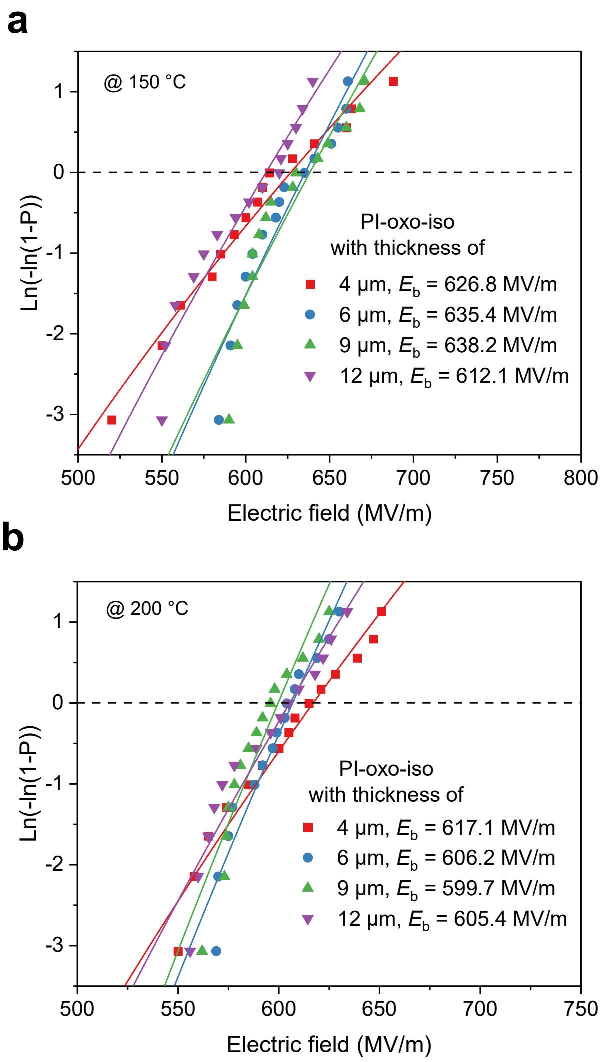

Supplementary Figure S50 Weibull distribution analysis of the PI-oxo-iso film with different thickness (4 μm, 6 μm, 9 μm, and 12 μm, respectively) at (a) 150 °C and (b) 200 °C.

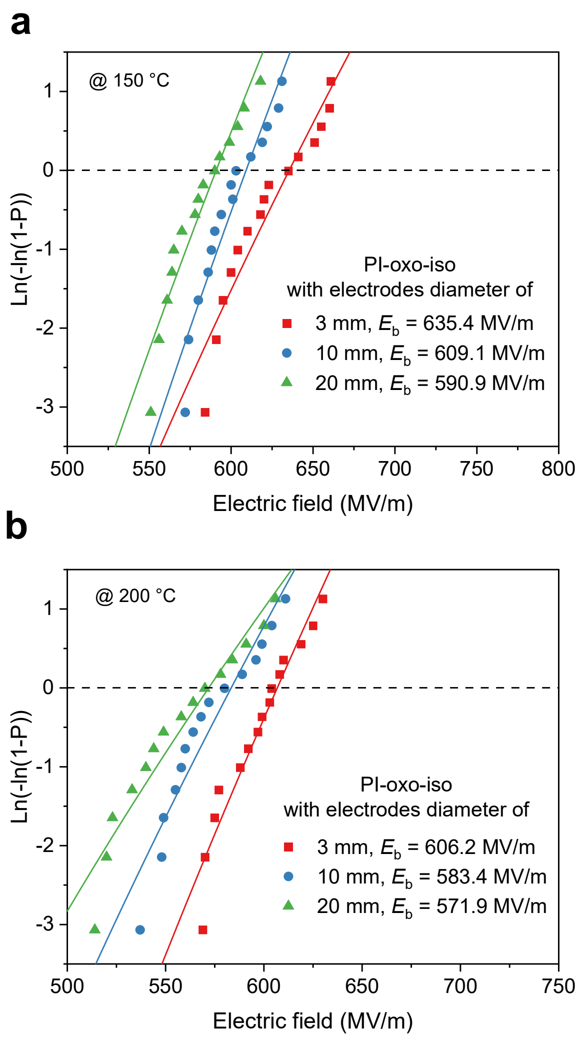

Supplementary Figure S51 Weibull distribution analysis of the PI-oxo-iso film with different electrode area (3 mm, 10 mm, and 20 mm diameter electrodes, respectively) at (a) 150 °C and (b) 200 °C.

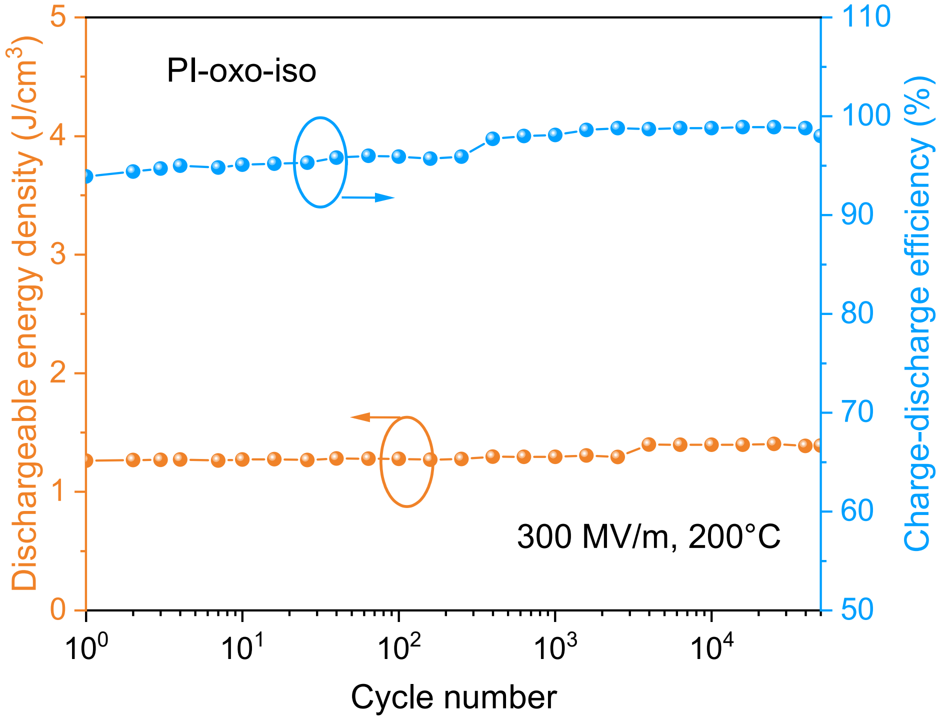

Supplementary Figure S52 Cyclic performance of PI-oxo-iso under 300 MV/m at 200 °C.

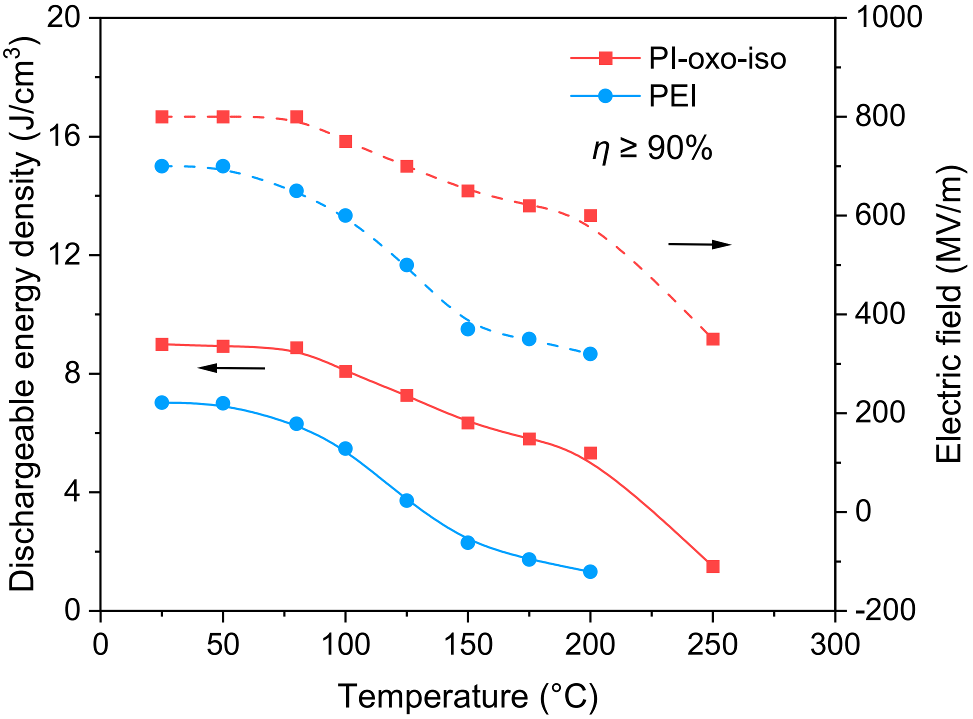

Supplementary Figure S53 Temperature-dependent electric field and discharged energy density of PI-oxo-iso and PEI under the precondition that discharge efficiency is higher than 90%. Considering the difference of glass transition temperature, the maximum test temperature of PEI and PI-oxo-iso is defined as 200 °C and 250 °C, respectively.

Section 5. Customized polymers at different temperatures

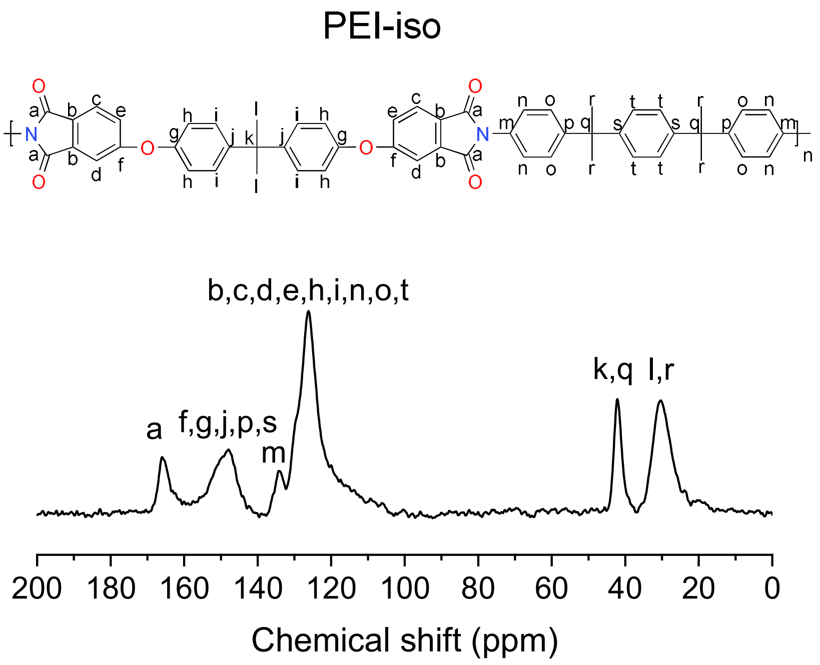

Supplementary Figure S54 ^{13}C NMR spectra of PEI-iso.

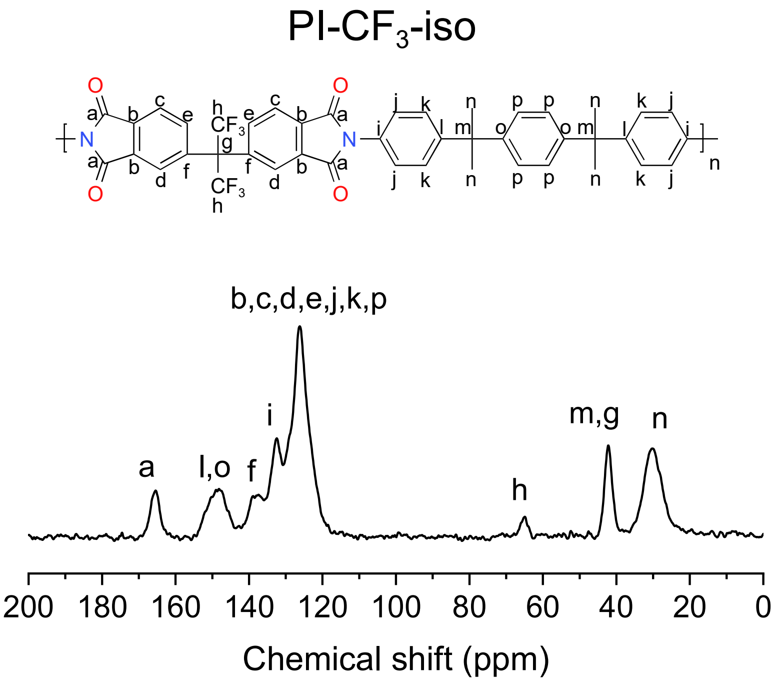

Supplementary Figure S55 ^{13}C NMR spectra of PI-CF_3-iso.

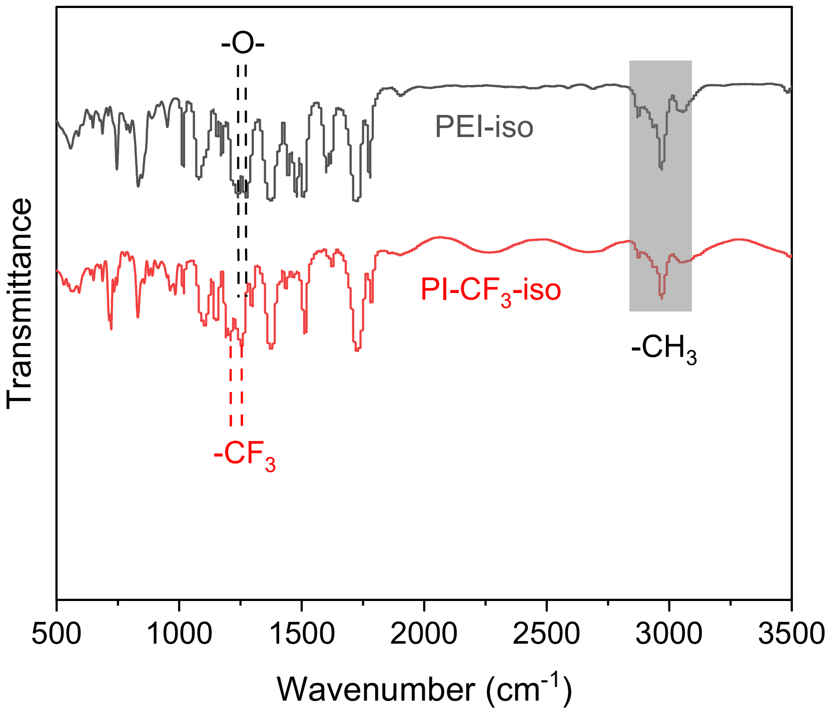

Supplementary Figure S56 ATR-FTIR spectra of PEI-iso and PI-CF_3-iso.

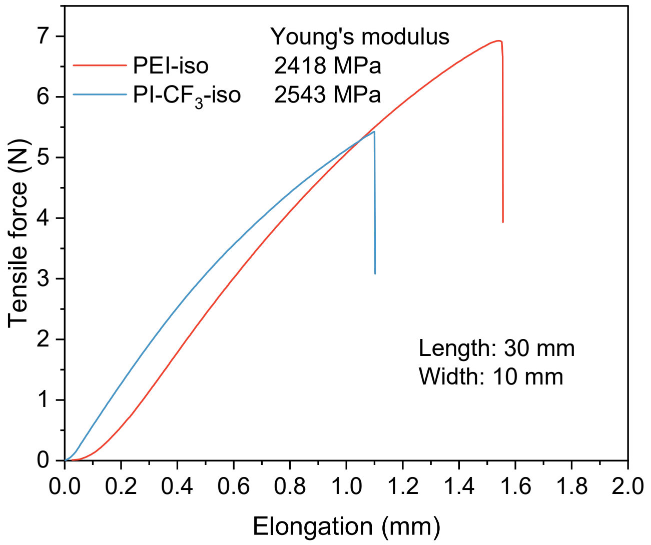

Supplementary Figure S57 Stress strain curve and Young's modulus of PEI-iso and PI-CF_3-iso.

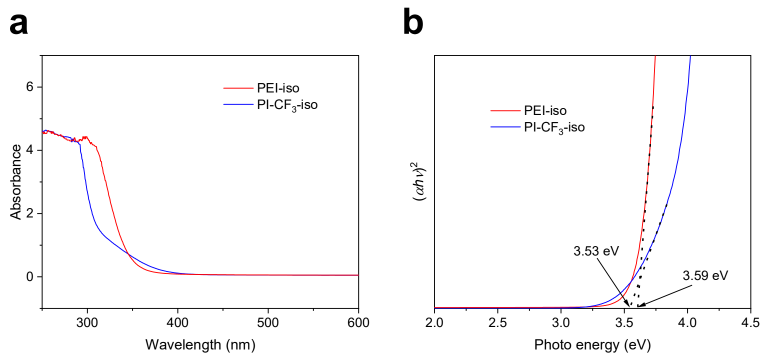

Supplementary Figure S58 (a) UV-vis absorption spectra and (b) Tacu plots of PEI-iso and PI-CF$_3$-iso.

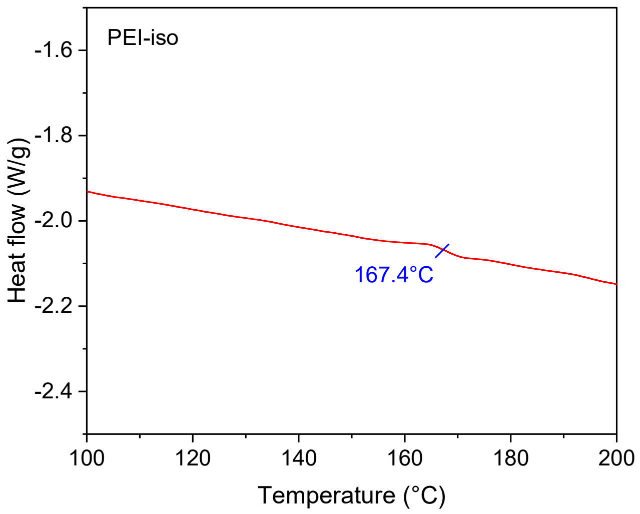

Supplementary Figure S59 Differential scanning calorimetric curves of PEI-iso.

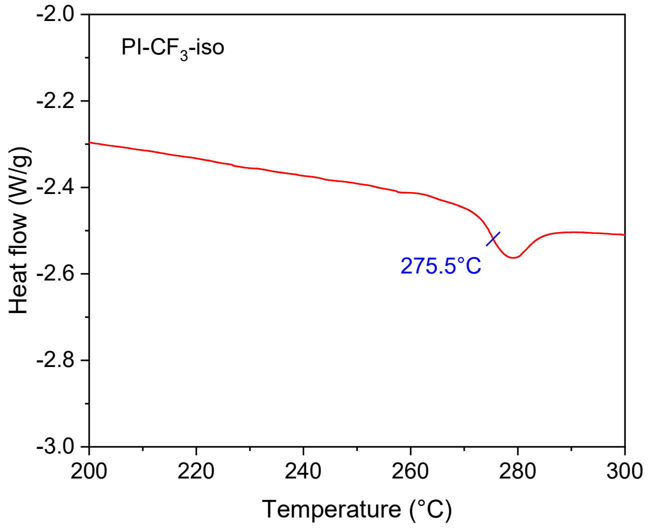

Supplementary Figure S60 Differential scanning calorimetric curves of PI-CF$_3$-iso.

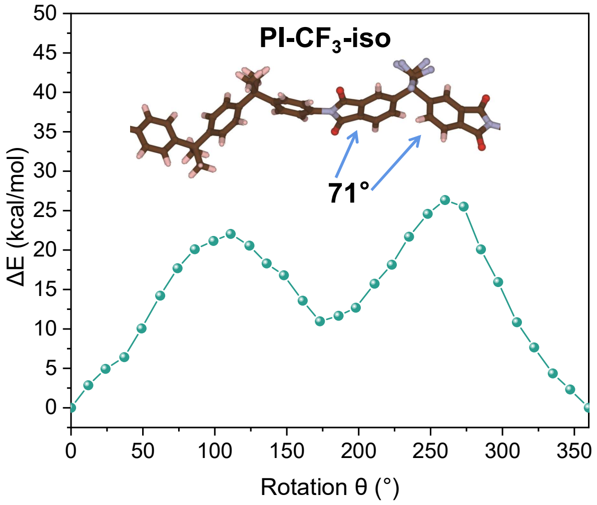

Supplementary Figure S61 The rotational energy barrier of benzene ring in PI-CF3-iso as a function of rotation angle.

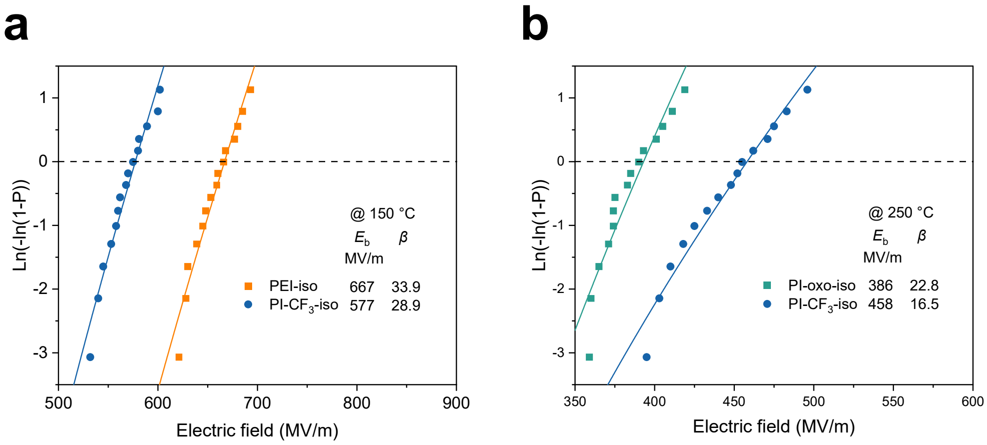

Supplementary Figure S62 Weibull distribution analysis of the PEI-iso, PI-oxo-iso and PI-CF3-iso at (a) 150 °C and (b) 250 °C.

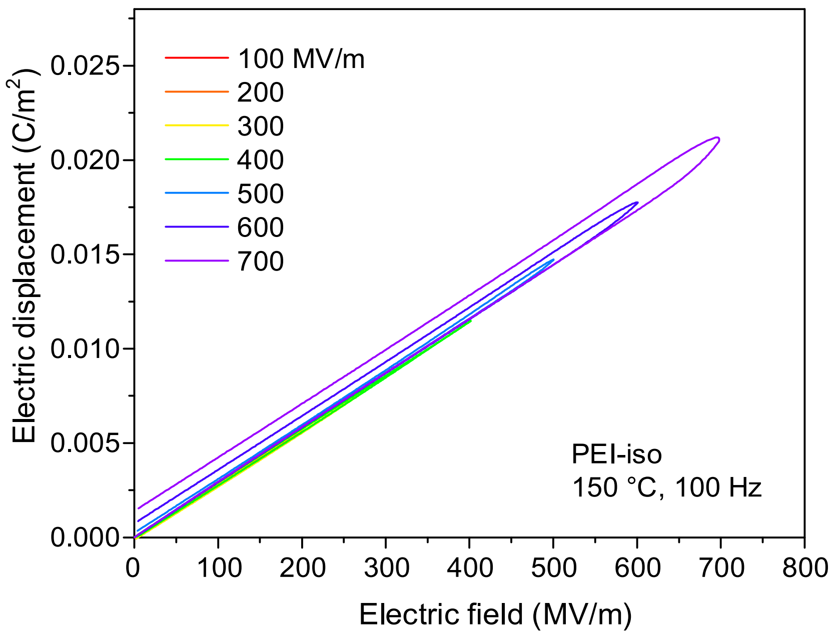

Supplementary Figure S63 Unipolar *D-E* loops of PEI-iso measured at 150 °C with 100 Hz.

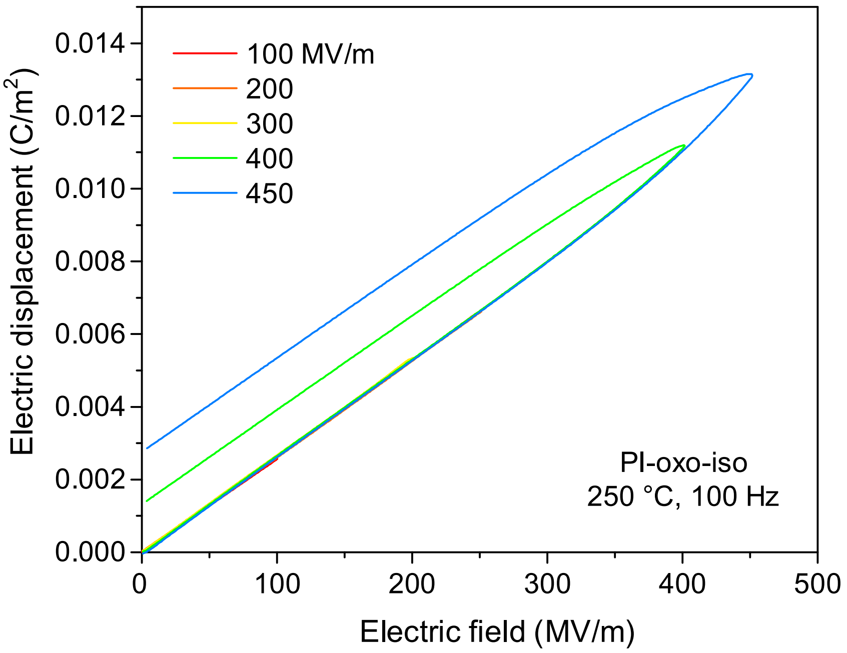

Supplementary Figure S64 Unipolar *D-E* loops of PI-oxo-iso measured at 250 °C with 100 Hz.

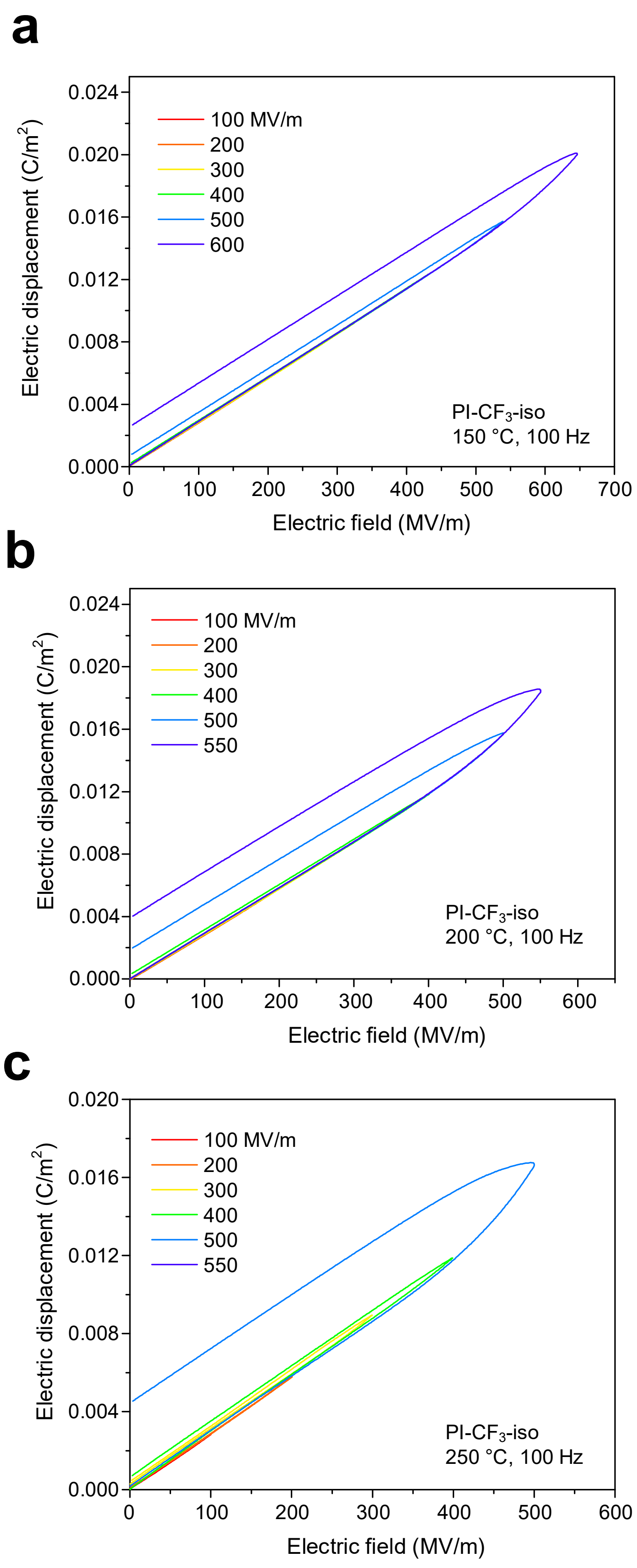

Supplementary Figure S65 Unipolar *D-E* loops of PI-CF_3-iso measured at (a) 150 °C with 100 Hz, (b) 200 °C with 100 Hz, (c) 250 °C with 100 Hz.

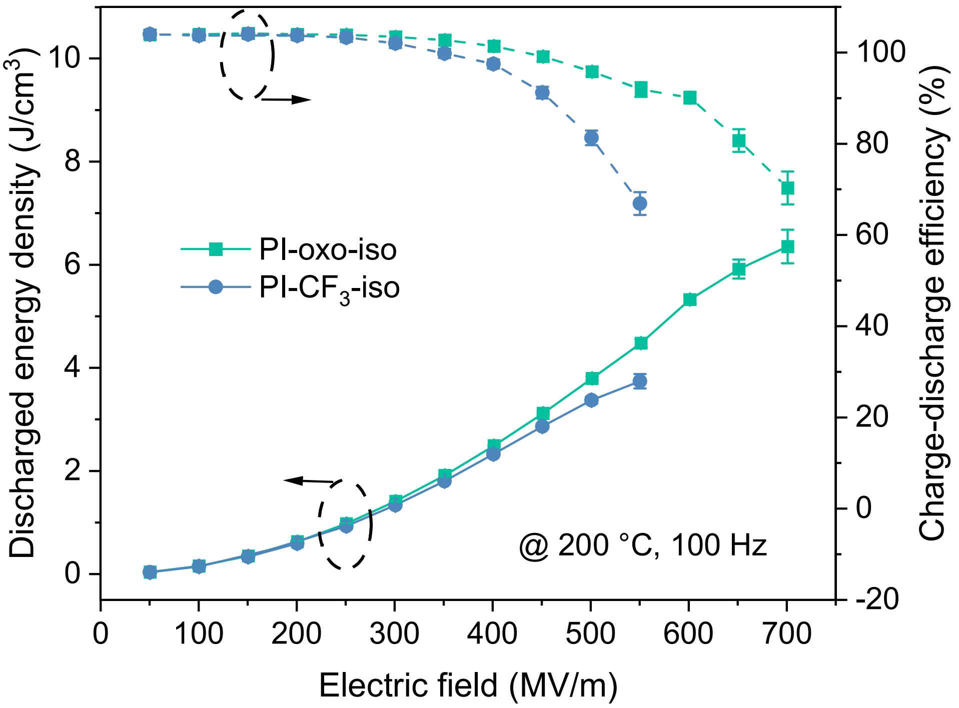

Supplementary Figure S66 Discharged energy density and charge-discharge efficiency as a function of electric field of PI-oxo-iso and PI-CF_3-iso at 200 °C. The average values and max–min error bars of the results were obtained from six parallel samples.

Supplementary Table S5. Specific amounts of the synthesis of the polymers.

Sample	Amount of diamine	Amount of dianhydride	Total mass
PI	160.2 mg (0.8 mmol)	174.5 mg (0.8 mmol)	334.7 mg
PI-m	160.2 mg (0.8 mmol)	174.5 mg (0.8 mmol)	334.7 mg
PI-methyl	158.6 mg (0.8 mmol)	174.5 mg (0.8 mmol)	333.1 mg
PI-ether	175.4 mg (0.6 mmol)	130.9 mg (0.6 mmol)	306.3 mg
PI-iso	206.7 mg (0.6 mmol)	130.9 mg (0.6 mmol)	337.6 mg
PI-oxo	120.1 mg (0.6 mmol)	186.1 mg (0.6 mmol)	306.2 mg
PI-oxo-M	119.0 mg (0.6 mmol)	186.1 mg (0.6 mmol)	305.1 mg
PI-oxo-iso	172.2 mg (0.5 mmol)	155.1 mg (0.5 mmol)	327.3 mg
PEI	54.1 mg (0.5 mmol)	260.2 mg (0.5 mmol)	314.3 mg
PEI-*p*	54.1 mg (0.5 mmol)	260.2 mg (0.5 mmol)	314.3 mg
PEI-*o*-M	61.1 mg (0.5 mmol)	260.2 mg (0.5 mmol)	321.3 mg
PI-cyc-iso	206.7 mg (0.6 mmol)	134.5 mg (0.6 mmol)	341.2 mg
PEI-iso	137.8 mg (0.4 mmol)	208.2 mg (0.4 mmol)	346.0 mg
PI-CF_3-iso	137.8 mg (0.4 mmol)	177.7 mg (0.4 mmol)	315.5 mg

Supplementary References

[1] Barth, S. et al. Current injection from a metal to a disordered hopping system. III. Comparison between experiment and Monte Carlo simulation. *Phys. Rev.* **60**, 8791 (1999).

[2] Kao, K. C. Dielectric Phenomena in Solids (Elsevier Academic Press, San Diego, 2004).

[3] McKeever, S. W. S. Thermoluminescence of Solids. Cambridge Univ Pr (1988).

[4] Mott, N. F. & Gurney, R. W. Electronic Processes in Ionic Crystals. Oxford University Press, London (1948).

[5] Tian, F. Q. et al. Theory of modified thermally stimulated current and direct determination of trap level distribution. *J. Electrostat.* **69**, 7-10 (2011).

[6] Li, H. et al. Scalable polymer nanocomposites with record high-temperature capacitive performance enabled by rationally designed nanostructured inorganic fillers. *Adv. Mater.* **31**, 1900875 (2019).

[7] Li, Q. et al. Flexible high-temperature dielectric materials from polymer nanocomposites. *Nature* **523**, 576-579 (2015).

[8] Yuan, C. et al. Polymer/molecular semiconductor all-organic composites for high-temperature dielectric energy storage. *Nat. Commun.* **11**, 3919 (2020).

[9] Niu, Y. J. et al. Significantly enhancing the discharge efficiency of sandwich-structured polymer dielectrics at elevated temperature by building carrier blocking interface. *Nano Energy* **97**, 107215 (2022).

[10] Zhang, K. Y. et al. Improving high-temperature energy storage performance of PI dielectric capacitor films through boron nitride interlayer. *Adv. Composit. Hybrid Ma.* **5**, 238-249 (2022).

[11] Dong, J. F. et al. A facile in situ surface-functionalization approach to scalable laminated high-temperature polymer dielectrics with ultrahigh capacitive performance. *Adv. Funct. Mater.* **31**, 2102644 (2021).

[12] Deshmukh, A. A. et al. Flexible polyolefin dielectric by strategic design of organic modules for harsh condition electrification. *Energy Environ. Sci.* **15**, 1307-1314 (2022).

[13] Cheng, S. et al. Polymer dielectrics sandwiched by medium-dielectric-constant nanoscale deposition layers for high-temperature capacitive energy storage. *Energy Storage Mater.* **42**, 445-453 (2021).

[14] Azizi, A. et al. High-performance polymers sandwiched with chemical vapor deposited hexagonal boron nitrides as scalable high-temperature dielectric materials. *Adv. Mater.* **29**, 1701864 (2017).

[15] Dong, J. F. et al. Enhancing high-temperature capacitor performance of polymer nanocomposites by adjusting the energy level structure in the micro-/meso-scopic interface region. *Nano Energy* **99**, 107314 (2022).

[16] Ren, W. B., et al. Scalable ultrathin all-organic polymer dielectric films for high-temperature capacitive energy storage. *Adv. Mater.* **34**, 2207421 (2022).
